# Modeling protein network evolution under genome duplication and domain shuffling

**DOI:** 10.1186/1752-0509-1-49

**Published:** 2007-11-13

**Authors:** Kirill Evlampiev, Hervé Isambert

**Affiliations:** 1 RNA dynamics and Biomolecular Systems Lab, CNRS UMR168, Institut Curie, Section de Recherche, 11 rue P. & M. Curie, 75005 Paris, France

## Abstract

**Background:**

Successive whole genome duplications have recently been firmly established in all major eukaryote kingdoms. Such *exponential *evolutionary processes must have largely contributed to shape the topology of protein-protein interaction (PPI) networks by outweighing, in particular, all *time-linear *network growths modeled so far.

**Results:**

We propose and solve a mathematical model of PPI network evolution under successive genome duplications. This demonstrates, from first principles, that evolutionary conservation and scale-free topology are intrinsically linked properties of PPI networks and emerge from *i) *prevailing *exponential *network dynamics under duplication and *ii) asymmetric divergence *of gene duplicates. While required, we argue that this asymmetric divergence arises, in fact, spontaneously at the level of protein-binding sites. This supports a refined model of PPI network evolution in terms of protein domains under exponential and asymmetric duplication/divergence dynamics, with multidomain proteins underlying the combinatorial formation of protein complexes. Genome duplication then provides a powerful source of PPI network innovation by promoting local rearrangements of multidomain proteins on a genome wide scale. Yet, we show that the overall conservation and topology of PPI networks are robust to extensive domain shuffling of multidomain proteins as well as to finer details of protein interaction and evolution. Finally, large scale features of *direct *and *indirect *PPI networks of *S. cerevisiae *are well reproduced numerically with only two adjusted parameters of clear biological significance (*i.e*. network effective growth rate and average number of protein-binding domains per protein).

**Conclusion:**

This study demonstrates the statistical consequences of genome duplication and domain shuffling on the conservation and topology of PPI networks over a broad evolutionary scale across eukaryote kingdoms. In particular, scale-free topologies of PPI networks, which are found to be robust to extensive shuffling of protein domains, appear to be a simple consequence of the conservation of protein-binding domains under asymmetric duplication/divergence dynamics in the course of evolution.

## Background

Gene duplication is considered the main evolutionary source of new protein functions [1]. Although long suspected [2,3], whole genome duplications have only been recently confirmed [4-12] through large scale comparisons of complete genomes.

Whole genome duplications are rare evolutionary transitions followed by random nonfunctionalization of many gene duplicates, resulting in characteristic reciprocal gene loss patterns [4,9,13], on time scales of about 100 MY (with large variations between genes, see Discussion). Whole genome duplications presumably provide unique opportunities to evolve many new functional genes at once through accretion of functional domains [14-20] from contiguous pseudogenes (or redundant genes) and may also promote speciation events by preventing genetic recombinations between close descendants with different reciprocal gene loss patterns [13,21].

Consecutive whole genome duplications (WGDs) have now been firmly established in all major eukaryote kingdoms within the last 300–500 MY, *i.e*. about 10–15% of life history.

WGDs have been more frequent in plants [22] due to their widespread polyploidy; for instance, there were 3 consecutive WGDs in the recent evolution of the flowering plants *Arabidopsis thaliana *[7] and *Populus trichocarpa *[23] while 4 WGDs can be identified in *Solanum *(potato), *Gossypium *(cotton) and *Brassica *genomes [22]. Overall, there were between 2 and 4 WGDs in plants in the last 300 MY and many extant species like *Solanum *(potato), *Glycine *(soybean) or *Saccharum *(sugarcane) have undergone a recent WGD and are still essentially pseudotetraploid plants with about twice as many gene loci as their close relatives lacking this recent WGD. They are living examples of the dramatic simultaneous changes a single WGD event produces on a genome. No other genome rearrangement is known to have a comparable immediate impact on the evolution of genomes (with the exception of endosymbiotic events).

Successive genome duplications have also occurred in animal genomes, even though most extant species are diploids. In vertebrates (chordates), there are, for instance, 4 consecutive WGDs between the seasquirt *Ciona intestinalis *and the common carp, *Cyprinus carpio*, with most tetrapods (including mammals) in between at +2WGDs from seasquirt and -2WGDs from carp and most bony fish at +3WGDs from seasquirt and -1WGDs from carp [11,12,24,25]. In fact, the common carp, *Cyprinus carpio*, and other bony fish from the salmonidae family (salmon, trout) as well as the amphibian *Xenopus laevis *and even the mammal *Tympanoctomys barrerae *(red vizcacha rat from Argentina [26]) are all pseudotetraploid vertebrates. [Constitutive tetraploidy is even occasionally observed in humans where it is responsible for 1 to 2% of early miscarriages but may lead, in rare cases, to liveborn infants reaching the age of two [27].

Amongst invertebrates, examples of polyploid species are also suspected or confirmed in most phyla, as in annelids (*e.g*., leeches [28]), flatworms (*e.g., Stenostomum *[29]), mollusks (*e.g*., Pacific oyster, *Crassostrea gigas *[30]) and in the major classes of arthropods, including insects (*e.g., Nabis pallidus *[31]), maxillopods (*e.g*., copepods [29]) and branchiopods (*e.g*., brine shrimp [32]). Finally, WGDs have also occured in protists; in particular, there were at least 3 consecutive WGDs in the ciliate *Paramecium tetraurelia *[33]. Other WGDs will likely be uncovered as more eukaryote sequences will become available.

Extrapolating from these 2 to 4 *consecutive *WGDs in the last 300–500 MY for typical eukaryote genomes, one roughly expects a few tens consecutive WGDs (or equivalent "doubling events") since the emergence of eukaryotes, if not the origin of life itself. [While WGDs do not seem readily traceable in extant prokaryote genomes, they cannot be ruled out either over long evolutionary time scales (*e.g*. > 500 MY). In fact, wildtype subpopulations of bacteria with stable diploid genomes are known to exist [34]. In addition, viable whole genome recombinants between different prokaryotes have also been successfully engineered [35].

These rare but dramatic evolutionary transitions due to whole genome duplications must have had major consequences on the long time scale evolution of large biological networks, such as protein-protein interaction (PPI) networks.

**Figure 1:**
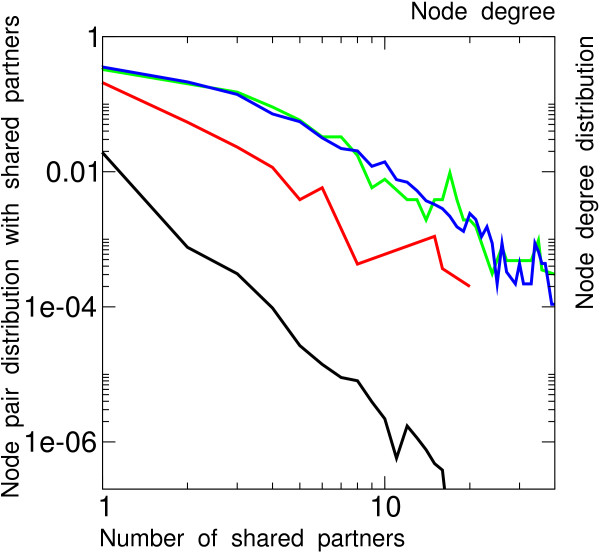
**Duplicated proteins from the 150 MY old WGD of *S. cerevisiae *share protein partners**. Distribution of duplicated (red) and random (black) node pairs versus number of shared partners. Node degree distribution of duplicated proteins (green) and all proteins of PPI network (blue).

**Figure 2:**
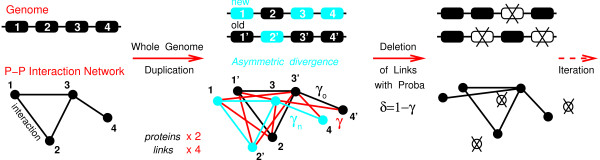
**Model of protein-protein interaction network evolution through whole genome duplication**. Whole genome duplication is followed by *asymmetric *divergence of protein duplicates with random distribution between genome copies (*e.g*. 1/1' vs 2/2'): "New" duplicates are left essentially free to accumulate neutral mutations with the likely outcome to become nonfunctional and eventually deleted unless some "new", *duplication-derived *interactions are selected; "Old" duplicates, on the other hand, are more constrained to conserve "old" interactions already present before duplication. The duplicated network with quadruplated links is graphically rearranged for convenience into old and new network copies (*e.g*. 2 and 2' duplicated nodes are swapped here). Links from the duplicated network are then kept with different probabilities *γ*_*i *_(0 ≤ *γ*_*i *_≤ 1) reflecting this asymmetric divergence between protein duplicates. An alternative model based on symmetric divergence of protein duplicates and random link "complementation" is illustrated in Fig. S1 and discussed in Supporting Information.

**Figure 3:**
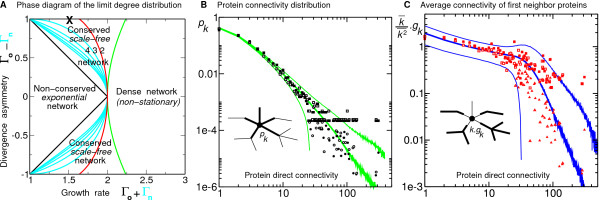
**Analytical and numerical results of PPI Network evolution under whole genome duplication**. **A**. Phase diagram for the limit degree distribution as a function of network exponential growth rate, Γ_o _+ Γ_n_, and asymmetric divergence of gene duplicates, Γ_o _- Γ_n_. In paricular, network conservation and scale-free topology are found to be intrinsically linked properties of PPI networks under genome duplication. Colored lines correspond to iso-exponent of scale-free degree distribution. All other regions of phase diagram are likely biologically irrelevant (see text). **B&C. **Comparison with protein *direct *physical interaction data for Yeast from BIND [38] and MIPS [39] databases: BIND (August 11, 2005 release), 4576 proteins, 9133 physical interactions, k¯
 MathType@MTEF@5@5@+=feaafiart1ev1aaatCvAUfKttLearuWrP9MDH5MBPbIqV92AaeXatLxBI9gBaebbnrfifHhDYfgasaacPC6xNi=xH8viVGI8Gi=hEeeu0xXdbba9frFj0xb9qqpG0dXdb9aspeI8k8fiI+fsY=rqGqVepae9pg0db9vqaiVgFr0xfr=xfr=xc9adbaqaaeGacaGaaiaabeqaaeqabiWaaaGcbaGafm4AaSMbaebaaaa@2D4A@ = 3.99, k2¯
 MathType@MTEF@5@5@+=feaafiart1ev1aaatCvAUfKttLearuWrP9MDH5MBPbIqV92AaeXatLxBI9gBaebbnrfifHhDYfgasaacPC6xNi=xH8viVGI8Gi=hEeeu0xXdbba9frFj0xb9qqpG0dXdb9aspeI8k8fiI+fsY=rqGqVepae9pg0db9vqaiVgFr0xfr=xfr=xc9adbaqaaeGacaGaaiaabeqaaeqabiWaaaGcbaWaa0aaaeaacqWGRbWAdaahaaWcbeqaaiabikdaYaaaaaaaaa@2E62@ = 106 (filled symbols) and MIPS (downloaded online April 20, 2006), 4153 proteins, 7417 physical interactions, k¯
 MathType@MTEF@5@5@+=feaafiart1ev1aaatCvAUfKttLearuWrP9MDH5MBPbIqV92AaeXatLxBI9gBaebbnrfifHhDYfgasaacPC6xNi=xH8viVGI8Gi=hEeeu0xXdbba9frFj0xb9qqpG0dXdb9aspeI8k8fiI+fsY=rqGqVepae9pg0db9vqaiVgFr0xfr=xfr=xc9adbaqaaeGacaGaaiaabeqaaeqabiWaaaGcbaGafm4AaSMbaebaaaa@2D4A@ = 3.57, k2¯
 MathType@MTEF@5@5@+=feaafiart1ev1aaatCvAUfKttLearuWrP9MDH5MBPbIqV92AaeXatLxBI9gBaebbnrfifHhDYfgasaacPC6xNi=xH8viVGI8Gi=hEeeu0xXdbba9frFj0xb9qqpG0dXdb9aspeI8k8fiI+fsY=rqGqVepae9pg0db9vqaiVgFr0xfr=xfr=xc9adbaqaaeGacaGaaiaabeqaaeqabiWaaaGcbaWaa0aaaeaacqWGRbWAdaahaaWcbeqaaiabikdaYaaaaaaaaa@2E62@ = 78.6 (open symbols). Squares correspond to raw data, while circles and triangles are statistically averaged with gaps in connectivity distribution for large *k *≥ 20, due to the finite size of Yeast PPI network. **B. **One-parameter fit of connectivity distribution data *p*_*k *_(corresponding to the "X" mark in **A.**, see text). Numerical connectivity distribution averaged over 10,000 network realizations (central green line). Numerical averages plus or minus two standard deviations (±2*σ*) are also displayed to show the predicted dispersions (upper and lower green lines) [Raw data (squares) do not fit within the mean ± 2*σ *curves for large *k *due to the finite size of Yeast PPI network]. The fitting parameter *γ *= 0.26 corresponds to an effective growth rate of 1 + 2*γ *= 1.52. **C. **One-parameter fit of average connectivity of first neighbor proteins *g*_*k *_[50] (*i.e. k.g*_*k *_sums connectivities of first neighbors from proteins of connectivity *k*). Numerical predictions averaged over 10,000 network realizations (central blue line). Numerical averages plus or minus two standard deviations are also displayed (upper and lower blue lines). Same fitting parameter value as in **B**, *γ *= 0.26. Note that *g*_*k *_is rescaled by k¯
 MathType@MTEF@5@5@+=feaafiart1ev1aaatCvAUfKttLearuWrP9MDH5MBPbIqV92AaeXatLxBI9gBaebbnrfifHhDYfgasaacPC6xNi=xH8viVGI8Gi=hEeeu0xXdbba9frFj0xb9qqpG0dXdb9aspeI8k8fiI+fsY=rqGqVepae9pg0db9vqaiVgFr0xfr=xfr=xc9adbaqaaeGacaGaaiaabeqaaeqabiWaaaGcbaGafm4AaSMbaebaaaa@2D4A@/k2¯
 MathType@MTEF@5@5@+=feaafiart1ev1aaatCvAUfKttLearuWrP9MDH5MBPbIqV92AaeXatLxBI9gBaebbnrfifHhDYfgasaacPC6xNi=xH8viVGI8Gi=hEeeu0xXdbba9frFj0xb9qqpG0dXdb9aspeI8k8fiI+fsY=rqGqVepae9pg0db9vqaiVgFr0xfr=xfr=xc9adbaqaaeGacaGaaiaabeqaaeqabiWaaaGcbaWaa0aaaeaacqWGRbWAdaahaaWcbeqaaiabikdaYaaaaaaaaa@2E62@ (as kgk¯
 MathType@MTEF@5@5@+=feaafiart1ev1aaatCvAUfKttLearuWrP9MDH5MBPbIqV92AaeXatLxBI9gBaebbnrfifHhDYfgasaacPC6xNi=xH8viVGI8Gi=hEeeu0xXdbba9frFj0xb9qqpG0dXdb9aspeI8k8fiI+fsY=rqGqVepae9pg0db9vqaiVgFr0xfr=xfr=xc9adbaqaaeGacaGaaiaabeqaaeqabiWaaaGcbaWaa0aaaeaacqWGRbWAcqWGNbWzdaWgaaWcbaGaem4AaSgabeaaaaaaaa@3025@ = k2¯
 MathType@MTEF@5@5@+=feaafiart1ev1aaatCvAUfKttLearuWrP9MDH5MBPbIqV92AaeXatLxBI9gBaebbnrfifHhDYfgasaacPC6xNi=xH8viVGI8Gi=hEeeu0xXdbba9frFj0xb9qqpG0dXdb9aspeI8k8fiI+fsY=rqGqVepae9pg0db9vqaiVgFr0xfr=xfr=xc9adbaqaaeGacaGaaiaabeqaaeqabiWaaaGcbaWaa0aaaeaacqWGRbWAdaahaaWcbeqaaiabikdaYaaaaaaaaa@2E62@ holds for each network realization); this rescales large *g*_*k *_fluctuations between network realizations, due to the divergence of k2¯
 MathType@MTEF@5@5@+=feaafiart1ev1aaatCvAUfKttLearuWrP9MDH5MBPbIqV92AaeXatLxBI9gBaebbnrfifHhDYfgasaacPC6xNi=xH8viVGI8Gi=hEeeu0xXdbba9frFj0xb9qqpG0dXdb9aspeI8k8fiI+fsY=rqGqVepae9pg0db9vqaiVgFr0xfr=xfr=xc9adbaqaaeGacaGaaiaabeqaaeqabiWaaaGcbaWaa0aaaeaacqWGRbWAdaahaaWcbeqaaiabikdaYaaaaaaaaa@2E62@ for *p*_*k *_~ *k*^-*_*-1 ^with 2 > *α *> 0 for the one-parameter model.

**Figure 4:**
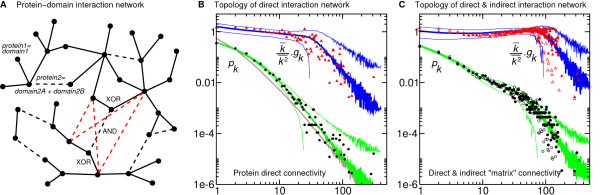
**Combining whole genome duplication and domain shuffling of multi-domain proteins**. **A**. Protein-domain interaction network. Nodes now correspond to single binding domains in a protein-domain interaction network (solid lines). Multi-binding-domain proteins are introduced through a new type of links corresponding to covalent peptide bonds between protein domains (black dashed lines). This provides a graphical framework to distinguish mutually exclusive, direct interactions ("XOR") between protein domains from cummulative, indirect interactions ("AND") within multi-protein complexes (red dashed lines). **B&C. **Comparison with protein direct & indirect interaction data for Yeast from BIND [38] database (**B&C **filled symbols, indirect interactions from [75,76]) and Ref [77] (**C **open symbols, see Supporting Information). Data are statistically averaged as in Fig. 3B&C to account for gaps in connectivities for large *k *≥ 20, due to the finite size of Yeast PPI network. **B. **Two-parameter fit of both direct connectivity distribution *p*_*k *_and average direct connectivity of first neighbor proteins *g*_*k *_[50] (see Fig. 3C and text). Numerical predictions are averaged over 1,000 network realizations (central green and blue lines). Numerical averages plus or minus two standard deviations are also displayed to show the predicted dispersions (upper and lower green and blue lines). The two adjusted parameters (*γ *= 0.1 and *λ *= 0.3) correspond to a network growth rate of 20% and an average of 1.5 protein-binding sites (domains) per protein. The connectivity distribution of the underlying single-domain network (corresponding to *γ *= 0.1 and *λ *= 0.0) is also displayed (brown line) to illustrate its relation to the full multi-domain protein network (see text). **C. **Two-parameter fit of both direct & indirect "matrix" connectivity distribution *p*_*k *_and average direct & indirect "matrix" connectivity of first neighbor proteins *g*_*k *_[50] (see text). Same two adjusted parameters (*γ *= 0.1 and *λ *= 0.3) as in **B **while a selection of indirect interactions is added up to a total of 28,000 direct and indirect interactions (see Supporting Information).

In this paper, we first discuss some experimental evidences (Fig. 1) and expected consequences of WGDs on the evolution of PPI networks. We then introduce a general model of PPI network evolution under WGD with *asymmetric *divergence of duplicated genes (Figs. 2 &3A). It is first compared to datasets of *direct *physical interactions from Yeast PPI network (Figs. 3B &3C) and also to an alternative model with *symmetric *protein divergence but random link "complementation" [36,37] (Additional file 1(Fig. S1)). We then redefine this initial asymmetric divergence model (Fig. 2) in terms of protein-binding domains (Figs. 4A &4B) to account for *indirect *protein-protein interaction within multi-protein complexes (Figs. 4A &4C) and study the robustness of PPI network topology against domain shuffling of multi-domain proteins.

## Results

### Effect of WGD on PPI network evolution

A direct experimental evidence for the effect of WGD on PPI network evolution is illustrated in Fig. 1. It concerns the baker yeast, *S. cerevisiae*, which has the best available PPI network dataset [38,39] and a well established WGD dating back about 150 MY [4,6,9]. About 90% of the initial pairs of duplicated proteins from this WGD have since then undergone reciprocal gene loss, leaving about 549 remaining pairs in the extant genome, amongst which 259 have both duplicated proteins included in the available PPI network [38]. The latter pairs of duplicated proteins are found to be about 20 times more likely to share some common protein partners as compared to randomly picked pairs of proteins, while their connectivity distribution is essentially the same as other interacting proteins in the PPI network, Fig. 1. This demonstrates that at least some of the duplicated interactions that were necessary present immediately after WGD have not been lost in the course of 150 MY of evolution, despites the divergence of the corresponding duplicated pairs and all their (initially) shared partners. The same trend has also been reported when considering protein pairs with a significant sequence homology [40]. This direct experimental evidence for the effect of WGD on PPI network evolution is even more compelling when considering protein pairs sharing more than one partner in the PPI network; for instance, duplicated pairs from this 150 MY-old WGD are about 1,000 times more likely to share 10 or more partners as compared to randomly picked pairs of the PPI network, Fig. 1.

From a more theoretical point of view and on longer evolutionary time scales (*e.g*. > 500 MY), we also expect that alternating WGDs and extensive gene deletions lead to *exponential *dynamics of PPI network evolution. In the long time limit, this should outweigh all *time-linear *dynamics that have been assumed in PPI network evolution models so far [36,41-45] (see, however, Discussion). In fact, the prevailing exponential dynamics of genome evolution is already clear from the wide distribution of genome sizes [1,3] and proliferation of repetitive elements [46]: it is hard to imagine that the 10^4^-fold span in lengths of eukaryote genomes could have solely arisen through time-linear increases (and decreases) in genome sizes. [There is even a 10^5^-fold span in genome lengths when including prokaryotes and 10^8^-fold including viruses].

### Overview of the model

We propose a simple model of PPI network evolution focussing on the effect of whole genome duplication (extensions to local or partial genome duplication are presented in ref [47] and confirm the conclusions of this paper, see also Discussion). In the present model, each time step *n *corresponds to a whole genome duplication and leads to a complete duplication of the PPI network, whereby each node is duplicated (×2) and each interaction quadruplated (×4) as depicted on Fig. 2[48]. Hence, the model considers discrete time steps corresponding to WGD events. Natural selection is then modeled *statistically*, that is regarless of specific evolutionary advantages, at the level of duplication-derived interactions (see Discussion). Concretely, links from the duplicated network are assumed to be stochastically preserved (or deleted) with different probabilities *γ*_*i *_(or *δ*_*i *_= 1 - *γ*_*i*_) reflecting the divergence of protein duplicates. In principles, these probabilities *γ*_*i *_might vary [47] at each WGD event and between different proteins, but we will focus in this paper on the simplest relevant model based on the asymmetric divergence of duplicated genes following genome duplication. For each pair of duplicated genes, one copy, referred to as the "old" duplicate, diverges more slowly and retains many of the interactions of the parent gene, while the other copy, referred to as the "new" duplicate, diverges more rapidly and looses many of its duplication-derived interactions. At each WGD steps, the asymmetry between "old" and "new" duplicates defines three interaction divergence parameters: *γ*_*o*_, the probability to preserve duplication-derived interactions between pairs of slowly diverging "old" duplicates; *γ*_*n*_, the probability to preserve duplication-derived interactions between pairs of rapidly diverging "new" duplicates and *γ*, the probability to preserve duplication-derived interactions between pairs involving one "old" and one "new" duplicates, see Fig. 2. In practice, interactions between slowly diverging "old" partners are much more likely to be preserved than those involving one or all the more two rapidly diverging "new" partners, *i.e*. 1 ≃ *γ*_*o *_≫ *γ *≫ *γ*_*n *_≃ 0. "Old" and especially "new" duplicates that loose all their interactions with previous partners are then eliminated from the PPI network, while the "old" and "new" labels of selected duplicates are eventually all reset (to "old") before the next WGD iteration. Hence, "old" and "new" labels are only *transient *notations reflecting the asymmetric divergence of duplicated pairs after each WGD event (see *Method*).

The PPI network evolution resulting from these successive WGDs is first solved *analytically *in the asymptotic limit of large PPI networks and then *numerically *for comparison with the available data on the yeast PPI network. Finally, an extension of this model is proposed to include the role of protein domains and their extensive shuffling between multidomain proteins over long evolutionary time scales.

### Modelling PPI network evolution under WGD

The interaction network is characterized at each WGD step *n *by its number of nodes with *k *neighbors Nk(n)
 MathType@MTEF@5@5@+=feaafiart1ev1aaatCvAUfKttLearuWrP9MDH5MBPbIqV92AaeXatLxBI9gBaebbnrfifHhDYfgasaacPC6xNi=xH8viVGI8Gi=hEeeu0xXdbba9frFj0xb9qqpG0dXdb9aspeI8k8fiI+fsY=rqGqVepae9pg0db9vqaiVgFr0xfr=xfr=xc9adbaqaaeGacaGaaiaabeqaaeqabiWaaaGcbaGaemOta40aa0baaSqaaiabdUgaRbqaaiabcIcaOiabd6gaUjabcMcaPaaaaaa@319B@ and its total number of links L(n)=∑k≥1kNk(n)/2
 MathType@MTEF@5@5@+=feaafiart1ev1aaatCvAUfKttLearuWrP9MDH5MBPbIqV92AaeXatLxBI9gBaebbnrfifHhDYfgasaacPC6xNi=xH8viVGI8Gi=hEeeu0xXdbba9frFj0xb9qqpG0dXdb9aspeI8k8fiI+fsY=rqGqVepae9pg0db9vqaiVgFr0xfr=xfr=xc9adbaqaaeGacaGaaiaabeqaaeqabiWaaaGcbaGaemitaW0aaWbaaSqabeaacqGGOaakcqWGUbGBcqGGPaqkaaGccqGH9aqpdaaeqaqaaiabdUgaRjabd6eaonaaDaaaleaacqWGRbWAaeaacqGGOaakcqWGUbGBcqGGPaqkaaGccqGGVaWlcqaIYaGmaSqaaiabdUgaRjabgwMiZkabigdaXaqab0GaeyyeIuoaaaa@4049@. Yet, we are not concerned by the evolutionary details of a particular network realization but rather by the statistical consequences of successive WGD events on the long evolutionary time scale of typical PPI networks. To this end, stochastic differences between possible PPI networks are averaged over all network realizations, and noted as ⟨Nk(n)
 MathType@MTEF@5@5@+=feaafiart1ev1aaatCvAUfKttLearuWrP9MDH5MBPbIqV92AaeXatLxBI9gBaebbnrfifHhDYfgasaacPC6xNi=xH8viVGI8Gi=hEeeu0xXdbba9frFj0xb9qqpG0dXdb9aspeI8k8fiI+fsY=rqGqVepae9pg0db9vqaiVgFr0xfr=xfr=xc9adbaqaaeGacaGaaiaabeqaaeqabiWaaaGcbaGaemOta40aa0baaSqaaiabdUgaRbqaaiabcIcaOiabd6gaUjabcMcaPaaaaaa@319B@⟩ for *k *≥ 0 and ⟨*L*^(*n*)^⟩. In addition, because evolutionary changes in the averages ⟨Nk(n)
 MathType@MTEF@5@5@+=feaafiart1ev1aaatCvAUfKttLearuWrP9MDH5MBPbIqV92AaeXatLxBI9gBaebbnrfifHhDYfgasaacPC6xNi=xH8viVGI8Gi=hEeeu0xXdbba9frFj0xb9qqpG0dXdb9aspeI8k8fiI+fsY=rqGqVepae9pg0db9vqaiVgFr0xfr=xfr=xc9adbaqaaeGacaGaaiaabeqaaeqabiWaaaGcbaGaemOta40aa0baaSqaaiabdUgaRbqaaiabcIcaOiabd6gaUjabcMcaPaaaaaa@319B@⟩ are coupled to one another for all node degrees *k *≥ 0, it is convenient to model the evolution of these averages ⟨Nk(n)
 MathType@MTEF@5@5@+=feaafiart1ev1aaatCvAUfKttLearuWrP9MDH5MBPbIqV92AaeXatLxBI9gBaebbnrfifHhDYfgasaacPC6xNi=xH8viVGI8Gi=hEeeu0xXdbba9frFj0xb9qqpG0dXdb9aspeI8k8fiI+fsY=rqGqVepae9pg0db9vqaiVgFr0xfr=xfr=xc9adbaqaaeGacaGaaiaabeqaaeqabiWaaaGcbaGaemOta40aa0baaSqaaiabdUgaRbqaaiabcIcaOiabd6gaUjabcMcaPaaaaaa@319B@⟩ by introducing a linear transform of ⟨Nk(n)
 MathType@MTEF@5@5@+=feaafiart1ev1aaatCvAUfKttLearuWrP9MDH5MBPbIqV92AaeXatLxBI9gBaebbnrfifHhDYfgasaacPC6xNi=xH8viVGI8Gi=hEeeu0xXdbba9frFj0xb9qqpG0dXdb9aspeI8k8fiI+fsY=rqGqVepae9pg0db9vqaiVgFr0xfr=xfr=xc9adbaqaaeGacaGaaiaabeqaaeqabiWaaaGcbaGaemOta40aa0baaSqaaiabdUgaRbqaaiabcIcaOiabd6gaUjabcMcaPaaaaaa@319B@⟩ in the form of a "generating function",

F(n)(x)=∑k≥0〈Nk(n)〉xk
 MathType@MTEF@5@5@+=feaafiart1ev1aaatCvAUfKttLearuWrP9MDH5MBPbIqV92AaeXatLxBI9gBaebbnrfifHhDYfgasaacPC6xNi=xI8qiVKYPFjYdHaVhbbf9v8qqaqFr0xc9vqFj0dXdbba91qpepeI8k8fiI+fsY=rqGqVepae9pg0db9vqaiVgFr0xfr=xfr=xc9adbaqaaeGacaGaaiaabeqaaeqabiWaaaGcbaGaemOray0aaWbaaSqabeaacqGGOaakcqWGUbGBcqGGPaqkaaGccqGGOaakcqWG4baEcqGGPaqkcqGH9aqpdaaeqbqaaiabgMYiHlabd6eaonaaDaaaleaacqWGRbWAaeaacqGGOaakcqWGUbGBcqGGPaqkaaGccqGHQms8cqWG4baEdaahaaWcbeqaaiabdUgaRbaaaeaacqWGRbWAcqGHLjYScqaIWaamaeqaniabggHiLdaaaa@473A@

which includes all nodes of the network according to their connectivity *k *≥ 0. Permanently disconnected nodes (*k *= 0) need, however, to be removed from the list of relevant nodes, as they correspond to proteins that have in fact lost all previous interactions and presumably their function, and are eventually eliminated from the genome. To this end, we redefine the graph size as, 〈N(n)〉=∑k≥1〈Nk(n)〉
 MathType@MTEF@5@5@+=feaafiart1ev1aaatCvAUfKttLearuWrP9MDH5MBPbIqV92AaeXatLxBI9gBaebbnrfifHhDYfgasaacPC6xNi=xH8viVGI8Gi=hEeeu0xXdbba9frFj0xb9qqpG0dXdb9aspeI8k8fiI+fsY=rqGqVepae9pg0db9vqaiVgFr0xfr=xfr=xc9adbaqaaeGacaGaaiaabeqaaeqabiWaaaGcbaGaeyykJeUaemOta40aaWbaaSqabeaacqGGOaakcqWGUbGBcqGGPaqkaaGccqGHQms8cqGH9aqpdaaeqaqaaiabgMYiHlabd6eaonaaDaaaleaacqWGRbWAaeaacqGGOaakcqWGUbGBcqGGPaqkaaGccqGHQms8aSqaaiabdUgaRjabgwMiZkabigdaXaqab0GaeyyeIuoaaaa@441C@, where 〈N0(n)〉
 MathType@MTEF@5@5@+=feaafiart1ev1aaatCvAUfKttLearuWrP9MDH5MBPbIqV92AaeXatLxBI9gBaebbnrfifHhDYfgasaacPC6xNi=xH8viVGI8Gi=hEeeu0xXdbba9frFj0xb9qqpG0dXdb9aspeI8k8fiI+fsY=rqGqVepae9pg0db9vqaiVgFr0xfr=xfr=xc9adbaqaaeGacaGaaiaabeqaaeqabiWaaaGcbaGaeyykJeUaemOta40aa0baaSqaaiabicdaWaqaaiabcIcaOiabd6gaUjabcMcaPaaakiabgQYiXdaa@34B7@ has been removed, and introduce a normalized generating function *p*^(*n*)^(*x*) for the mean degree distribution,

p(n)(x)=∑k≥1pk(n)xk,wherepk(n)=〈Nk(n)〉〈N(n)〉
 MathType@MTEF@5@5@+=feaafiart1ev1aaatCvAUfKttLearuWrP9MDH5MBPbIqV92AaeXatLxBI9gBaebbnrfifHhDYfgasaacPC6xNi=xI8qiVKYPFjYdHaVhbbf9v8qqaqFr0xc9vqFj0dXdbba91qpepeI8k8fiI+fsY=rqGqVepae9pg0db9vqaiVgFr0xfr=xfr=xc9adbaqaaeGacaGaaiaabeqaaeqabiWaaaGcbaqbaeqabeWaaaqaaiabdchaWnaaCaaaleqabaGaeiikaGIaemOBa4MaeiykaKcaaOGaeiikaGIaemiEaGNaeiykaKIaeyypa0ZaaabuaeaacqWGWbaCdaqhaaWcbaGaem4AaSgabaGaeiikaGIaemOBa4MaeiykaKcaaOGaemiEaG3aaWbaaSqabeaacqWGRbWAaaaabaGaem4AaSMaeyyzImRaeGymaedabeqdcqGHris5aOGaeiilaWcabaGaee4DaCNaeeiAaGMaeeyzauMaeeOCaiNaeeyzaugabaGaemiCaa3aa0baaSqaaiabdUgaRbqaaiabcIcaOiabd6gaUjabcMcaPaaakiabg2da9KqbaoaalaaabaGaeyykJeUaemOta40aa0baaeaacqWGRbWAaeaacqGGOaakcqWGUbGBcqGGPaqkaaGaeyOkJepabaGaeyykJeUaemOta40aaWbaaeqabaGaeiikaGIaemOBa4MaeiykaKcaaiabgQYiXdaaaaaaaa@64FE@

The use of generating functions is a standard method [49] that enables to characterize distributions ⟨Nk(n)
 MathType@MTEF@5@5@+=feaafiart1ev1aaatCvAUfKttLearuWrP9MDH5MBPbIqV92AaeXatLxBI9gBaebbnrfifHhDYfgasaacPC6xNi=xH8viVGI8Gi=hEeeu0xXdbba9frFj0xb9qqpG0dXdb9aspeI8k8fiI+fsY=rqGqVepae9pg0db9vqaiVgFr0xfr=xfr=xc9adbaqaaeGacaGaaiaabeqaaeqabiWaaaGcbaGaemOta40aa0baaSqaaiabdUgaRbqaaiabcIcaOiabd6gaUjabcMcaPaaaaaa@319B@⟩ and *p*_*k *_from their successive moments, *e.g*. ∑k≥0〈kjNk(n)〉
 MathType@MTEF@5@5@+=feaafiart1ev1aaatCvAUfKttLearuWrP9MDH5MBPbIqV92AaeXatLxBI9gBaebbnrfifHhDYfgasaacPC6xNi=xH8viVGI8Gi=hEeeu0xXdbba9frFj0xb9qqpG0dXdb9aspeI8k8fiI+fsY=rqGqVepae9pg0db9vqaiVgFr0xfr=xfr=xc9adbaqaaeGacaGaaiaabeqaaeqabiWaaaGcbaWaaabeaeaacqGHPms4cqWGRbWAdaahaaWcbeqaaiabdQgaQbaakiabd6eaonaaDaaaleaacqWGRbWAaeaacqGGOaakcqWGUbGBcqGGPaqkaaGccqGHQms8aSqaaiabdUgaRjabgwMiZkabicdaWaqab0GaeyyeIuoaaaa@3E11@ via the successive derivatives of their generating functions, *e.g*. ∂xjF(n)(x)
 MathType@MTEF@5@5@+=feaafiart1ev1aaatCvAUfKttLearuWrP9MDH5MBPbIqV92AaeXatLxBI9gBaebbnrfifHhDYfgasaacPC6xNi=xH8viVGI8Gi=hEeeu0xXdbba9frFj0xb9qqpG0dXdb9aspeI8k8fiI+fsY=rqGqVepae9pg0db9vqaiVgFr0xfr=xfr=xc9adbaqaaeGacaGaaiaabeqaaeqabiWaaaGcbaGaeyOaIy7aa0baaSqaaiabdIha4bqaaiabdQgaQbaakiabdAeagnaaCaaaleqabaGaeiikaGIaemOBa4MaeiykaKcaaOGaeiikaGIaemiEaGNaeiykaKcaaa@37D4@, *j *≥ 1 (see Methods). While the node degree distributions *N*_*k *_and *p*_*k *_are purely local characteristics of networks, the use of generating functions can, in fact, be generalized [47] to other, possibly non local features of interest, such as the average connectivity of first neighbors *g*_*k *_[50], introduced below.

### Asymmetric divergence of duplicated proteins

In the following, we consider a general model of PPI network evolution under WGD which allows for asymmetric divergence of duplicated proteins, Fig. 2. Symmetric divergence of duplicate proteins corresponds to a particular case of divergence with vanishing asymmetry and is discussed in the Supporting Information in the context of an alternative model based on symmetric duplication-divergence processes with link "complementation" [36,37].

Actually, asymmetric divergence between duplicated genes is well supported by the reciprocal gene loss patterns arising after WGD [4,6,9]; this demonstrates that many, if not most, of the initially duplicated genes are eventually retained as single genes in the duplicated genome, reflecting clearly the asymmetric fate of duplicated genes after WGD (see, however, Discussion). Indeed, while duplicated genes are initially equivalent and experience, at first, the same functional constraints [51], their divergence becomes eventually asymmetric [52-54]. This occurs as one duplicate is more constrained to retain "old" interactions, while the other duplicate is less constrained and thus accumulates more mutations with the likely outcome to become nonfunctional by loosing all its duplication-derived interactions, unless some of them are eventually retained by selection. Note that the only interaction changes considered in this model are deletions of duplication-derived interactions (*e.g*. interactions arising from horizontal gene transfer are more characteristic of prokaryote evolution [55] and neglected here [45]). As outlined in the model overview above, divergence asymmetry is introduced by assigning different evolutionary parameters *γ*_o _and *γ*_n _in between "old" or "new" duplicated nodes corresponding to a larger and lower chance to conserve instances of their parent-node interactions, Fig. 2. Duplication-derived interactions arising between different "old" and "new" duplicates are retained with probability *γ*. Note that "old" and "new" labels in Fig. 2 refer to the asymmetric conservation and fate of duplicates after WGD (and *not *to specific genome copies). Functionalization patterns of duplicated genes are further discussed in additional file 1.

We have solved this mathematical model of PPI network evolution under WGD illustrated in Fig. 2. The theoretical approach detailed in Methods relies on asymptotic methods applied to a functional recurrence relating successive normalized generating functions *p*^(*n*)^(*x*) of the PPI network degree distribution, Eq. 2. We outline here, from a biological perspective, the main conclusions of this exact analytical approach. The main results only depend on the following two combinations of evolutionary parameters, Γ_o _= *γ*_o _+ *γ *and Γ_n _= *γ*_n _+ *γ*, which correspond to the average rates of connectivity change between successive WGDs, *k *→ *k*Γ_i_, for each type of duplicates, i = o, n. We assume Γ_o _≥ Γ_n _by definition of the more conserved ("old") and less conserved ("new") duplicates, respectively. Hence, the connectivity of the most conserved duplicates decreases or increases as kΓom
 MathType@MTEF@5@5@+=feaafiart1ev1aaatCvAUfKttLearuWrP9MDH5MBPbIqV92AaeXatLxBI9gBaebbnrfifHhDYfgasaacPC6xNi=xH8viVGI8Gi=hEeeu0xXdbba9frFj0xb9qqpG0dXdb9aspeI8k8fiI+fsY=rqGqVepae9pg0db9vqaiVgFr0xfr=xfr=xc9adbaqaaeGacaGaaiaabeqaaeqabiWaaaGcbaGaem4AaSMaeu4KdC0aa0baaSqaaiabb+gaVbqaaiabd2gaTbaaaaa@318F@ under *m *successive WGDs: the case Γ_o _< 1 corresponds to an exponential decrease of connectivity and eventual disappearence of any given node of the network. By contrast, the case Γ_o _> 1 corresponds to a connectivity increase of the "old" duplicate descents and, hence, to an overall conservation of the PPI network in the course of evolution under WGDs, see below and Discussion. Strikingly, it can be shown that this simple criteria on Γ_o _governs not only the evolutionary conservation but also the topology of the emerging PPI networks under WGDs, see Methods for detailed proof. The different evolutionary regimes and asymptotic degree distributions, *p*_*k*_, are summarized in the phase diagram Fig. 3A in the plane (Γ_o _+ Γ_n_,Γ_o _- Γ_n_). Each axis of this phase diagram has a simple, biologically relevant interpretation: Γ_o _+ Γ_n _is the global growth rate of the network in terms of number of interactions (Γ_o _+ Γ_n _> 1 to ensure a growing network) and Γ_o _- Γ_n _corresponds to the divergence asymmetry between duplicated proteins. We outline here the two main evolutionary regimes of the model and discuss their biological relevance (see Methods for proof details).

#### • Non-conserved, exponential regime

The case Γ_o _< 1 (and Γ_n _< 1) implies an exponentially decreasing degree distribution, *p*_*k *_∝ exp(-*μk*) for large *k *≫ 1, corresponding to a regular, infinitely derivable generating function, *p*(*x*). From an evolutionary perspective, we find that this exponential topology arises while the links emerging from each node (Fig. 2) are more likely lost than duplicated at each round of global duplication (as Γ_i _= *γ *+ *γ*_i _< 1 is equivalent to *δδ*_i _> *γγ*_i_). This implies that most nodes eventually disappear, and with them all traces of network evolution, after just a few rounds of global duplication. The network topology is *not *conserved, as anticipated above, but instead continuously renewed from duplication of the (few) most connected nodes. From a speciation perspective, this implies that all nodes of a given PPI network realization are eventually more closely related to one another than to any other node of a different PPI network realization, *i.e*. from a different species. Clearly, this class of evolutionary non-conserved PPI networks doest not appear to be biologically relevant, given the typical degree of conservation between orthologous proteins across living kingdoms. As a consequence, we can also conclude from the phase diagram Fig. 3A that exponential PPI networks arising through genome duplication would necessary correspond to non-conserved networks and would thus be presumably irrelevant from a biological perspective. This result actually holds, beyond genome duplication, for evolutionary duplication-divergence dynamics at *any *genomic scale (from single gene to whole genome) and even with *variations *in all evolutionary parameters {γi(n)}
 MathType@MTEF@5@5@+=feaafiart1ev1aaatCvAUfKttLearuWrP9MDH5MBPbIqV92AaeXatLxBI9gBaebbnrfifHhDYfgasaacPC6xNi=xH8viVGI8Gi=hEeeu0xXdbba9frFj0xb9qqpG0dXdb9aspeI8k8fiI+fsY=rqGqVepae9pg0db9vqaiVgFr0xfr=xfr=xc9adbaqaaeGacaGaaiaabeqaaeqabiWaaaGcbaGaei4EaShcciGae83SdC2aa0baaSqaaiabbMgaPbqaaiabcIcaOiabd6gaUjabcMcaPaaakiabc2ha9baa@3528@ at each duplication-divergence process *n*, see Discussion. Hence, only non-exponential topologies of PPI networks are likely to be observed in nature. This corresponds to the second regime discussed below.

#### • Conserved, scale-free regime

The case Γ_o _> 1 > Γ_n _implies a "scale-free" topology with a power law decrease of the node degree distribution *p*_*k *_∝ *k*^-*α*-1^, for large *k *≫ 1. This corresponds to a singular, non-infinitely derivable generating function, *p*(*x*), with the following asymptotic expansion in the vicinity of *x *= 1,

*p*(*x*) = 1 - *A*_1_(1 - *x*) - ... - *A*_*r*_(1 - *x*)^*r *^- *A*_*α *_(1 - *x*)^*α *^- ...

where *r *≥ 1 is an integer and *α *> 1 the solution of the following characteristic equation (with *r *≤ *α *<*r *+ 1),

Γnα+Γoα=Γn+Γo
 MathType@MTEF@5@5@+=feaafiart1ev1aaatCvAUfKttLearuWrP9MDH5MBPbIqV92AaeXatLxBI9gBaebbnrfifHhDYfgasaacPC6xNi=xI8qiVKYPFjYdHaVhbbf9v8qqaqFr0xc9vqFj0dXdbba91qpepeI8k8fiI+fsY=rqGqVepae9pg0db9vqaiVgFr0xfr=xfr=xc9adbaqaaeGacaGaaiaabeqaaeqabiWaaaGcbaGaeu4KdC0aa0baaSqaaiabb6gaUbqaaGGaciab=f7aHbaakiabgUcaRiabfo5ahnaaDaaaleaacqqGVbWBaeaacqWFXoqyaaGccqGH9aqpcqqHtoWrdaWgaaWcbaGaeeOBa4gabeaakiabgUcaRiabfo5ahnaaBaaaleaacqqGVbWBaeqaaaaa@3E2B@

When Γnr+Γor=Γn+Γo
 MathType@MTEF@5@5@+=feaafiart1ev1aaatCvAUfKttLearuWrP9MDH5MBPbIqV92AaeXatLxBI9gBaebbnrfifHhDYfgasaacPC6xNi=xH8viVGI8Gi=hEeeu0xXdbba9frFj0xb9qqpG0dXdb9aspeI8k8fiI+fsY=rqGqVepae9pg0db9vqaiVgFr0xfr=xfr=xc9adbaqaaeGacaGaaiaabeqaaeqabiWaaaGcbaGaeu4KdC0aa0baaSqaaiabb6gaUbqaaiabdkhaYbaakiabgUcaRiabfo5ahnaaDaaaleaacqqGVbWBaeaacqWGYbGCaaGccqGH9aqpcqqHtoWrdaWgaaWcbaGaeeOBa4gabeaakiabgUcaRiabfo5ahnaaBaaaleaacqqGVbWBaeqaaaaa@3D77@ for exactly some integer *r *≥ 1 the last term in Eq. 3 should be replaced by (1 - *x*)^*r *^ln(1 - *x*), and the limit degree distribution decreases like *k*^-*r*-1 ^(see "exponent" lines in Fig. 3A for *α *+ 1 = 2, 3, 4, ...). Hence, from an evolutionary perspective, we find that scale-free degree distributions emerge under successive, global network duplications only if the "old" node copies have their links more likely duplicated than lost at each round of global duplication (as Γ_o _= *γ *+ *γ*_o _> 1 is equivalent to *γγ*_o _> *δδ*_o_). Thus, "old" nodes statistically keep on increasing their connectivity once they have emerged as "new" nodes by duplication. This implies that most nodes and their surrounding links are conserved *throughout *the evolution process, thereby ensuring that local topologies of previous networks remain embedded in subsequent networks. Hence, the evolutionary conservation and scale-free topology of PPI networks appear intrinsically linked under genome duplication. Evolutionary conservation, which is a fundamental property of proteins and PPI networks (see *e.g*. Fig. 1) is shown to necessary lead to scale-free PPI network topologies. It is, in fact, a very general and fundamental result that is not sensitive to *variations *in the model parameters {γi(n)}
 MathType@MTEF@5@5@+=feaafiart1ev1aaatCvAUfKttLearuWrP9MDH5MBPbIqV92AaeXatLxBI9gBaebbnrfifHhDYfgasaacPC6xNi=xH8viVGI8Gi=hEeeu0xXdbba9frFj0xb9qqpG0dXdb9aspeI8k8fiI+fsY=rqGqVepae9pg0db9vqaiVgFr0xfr=xfr=xc9adbaqaaeGacaGaaiaabeqaaeqabiWaaaGcbaGaei4EaShcciGae83SdC2aa0baaSqaaiabbMgaPbqaaiabcIcaOiabd6gaUjabcMcaPaaakiabc2ha9baa@3528@ on the evolutionary time scale *n *and also holds for duplication-divergence events at *any *genomic scale from single gene to whole genome duplication (see Discussion). In other words, scale-free topologies of PPI networks appear to be a simple consequence of the evolutionary conservation of PPI networks and their underlying proteins.

In summary, whole genome duplication with asymmetric divergence of duplicated proteins leads to the emergence of two main classes of PPI networks : *i) *PPI networks with an exponential degree distribution and without protein nor topology evolutionary conservation and *ii) *PPI networks with a scale-free limit degree distribution and protein conservation together with at least some local topology conservation. All other evolution scenarios are unlikely to model biologically relevant cases; they correspond either to an exponential disappearance of the whole PPI network (*i.e*. if Γ_n _+ Γ_o _< 1) or to an exponential shift of *all *proteins towards higher and higher connectivities, *i.e*. dense regime in Fig. 3A, see Methods and [47]. Note, in particular, that the evolution of PPI networks with *symmetric *divergence under WGD, *i.e*. Γ_o _- Γ_n _= 0 in Fig. 3A, *cannot *lead to biologically relevant, conserved PPI networks with scale-free topology; Indeed, WGD followed by symmetric divergence of duplicated genes leads either to non-conserved exponential PPI networks (for 1 < Γ_o _+ Γ_n _< 2) or to nonstationary dense PPI networks (for 2 < Γ_o _+ Γ_n_). Besides, the same conclusion applies for an alternative model of PPI network evolution under WGD and "link complementation", see additional file 1. Hence, asymmetric divergence of duplicated genes under WGD is required to obtain a (non-dense) conserved PPI networks. Yet, we will argue, below, that such divergence asymmetry arises, in fact, spontaneously at the level of protein-binding *domains*. This will support a refined model of PPI network evolution in terms of protein domains rather than entire proteins.

### Fitting PPI network data with a one-parameter model

Scale-free degree distributions have been widely reported for large biological networks and other exponentially growing networks like the WWW. We showed in the previous discussion that scale-free limit degree distributions require an asymmetric divergence of duplicated proteins (Γ_o _- Γ_n _= *γ*_o _- *γ*_n _> 0) which corresponds to the probability difference between conservation of old interactions (*γ*_o_) and coevolution of new binding sites (*γ*_n_). The expected range of parameters for actual biological networks is 1 ≃ *γ*_o _≫ *γ *≫ *γ*_n _≃ 0; In particular, the most conservative (*γ*_o _= 1) and least correlated (*γ*_n _= 0) evolution scenario corresponds to the strongest divergence asymmetry between duplicated proteins (Γ_o _- Γ_n _= 1, upper border on Fig. 3A). The condition *γ*_o _= 1 ensures that not only local but also global topologies of all previous networks remain embedded in all subsequent networks. This model is effectively a one-parameter model (*γ*) for PPI network evolution through whole genome duplication. It converges towards a stationary scale-free limit degree distribution *p*_*k *_~ *k*^-*α*-1 ^with 0 <*α *< 2 for 0 <*γ *< (5
 MathType@MTEF@5@5@+=feaafiart1ev1aaatCvAUfKttLearuWrP9MDH5MBPbIqV92AaeXatLxBI9gBaebbnrfifHhDYfgasaacPC6xNi=xH8viVGI8Gi=hEeeu0xXdbba9frFj0xb9qqpG0dXdb9aspeI8k8fiI+fsY=rqGqVepae9pg0db9vqaiVgFr0xfr=xfr=xc9adbaqaaeGacaGaaiaabeqaaeqabiWaaaGcbaWaaOaaaeaacqaI1aqnaSqabaaaaa@2CE6@ - 1)/2 and generates non-stationary dense networks for (5
 MathType@MTEF@5@5@+=feaafiart1ev1aaatCvAUfKttLearuWrP9MDH5MBPbIqV92AaeXatLxBI9gBaebbnrfifHhDYfgasaacPC6xNi=xH8viVGI8Gi=hEeeu0xXdbba9frFj0xb9qqpG0dXdb9aspeI8k8fiI+fsY=rqGqVepae9pg0db9vqaiVgFr0xfr=xfr=xc9adbaqaaeGacaGaaiaabeqaaeqabiWaaaGcbaWaaOaaaeaacqaI1aqnaSqabaaaaa@2CE6@ - 1)/2 <*γ *< 1 [47]. We used this one-parameter model to fit both the degree distribution (Fig. 3B) and the average connectivity of first neighbors (Fig. 3C) for *direct *physical interaction data of *S. cerevisiae *taken from two databases, BIND [38] and MIPS [39]. BIND data mainly comes from high throughput two-hybrid techniques, while MIPS data is primarily based on hand curated, litterature references (with presumably fewer nonspecific spurious interactions). The predicted asymptotic regime is in fact approached for *k *≤ 20 due to the finite size of Yeast PPI network. Note, in particular, that *both *scale-free degree distribution (Fig. 3B) and protein hub repulsion (so-called network "disassortativity" [42,50], Fig. 3C) are *simultaneously *predicted with a single fitting parameter *γ *= 0.26. This corresponds to a fixed growth rate Γ_o _+ Γ_n _= 1 + 2*γ *= 1.52 (*i.e*. the number of links and nodes increases by 52% at each global duplication).

Adding and removing up to 30% of links randomly, or drawing *γ *from a uniform distribution between 0 and 0.52 (with average γ¯
 MathType@MTEF@5@5@+=feaafiart1ev1aaatCvAUfKttLearuWrP9MDH5MBPbIqV92AaeXatLxBI9gBaebbnrfifHhDYfgasaacPC6xNi=xH8viVGI8Gi=hEeeu0xXdbba9frFj0xb9qqpG0dXdb9aspeI8k8fiI+fsY=rqGqVepae9pg0db9vqaiVgFr0xfr=xfr=xc9adbaqaaeGacaGaaiaabeqaaeqabiWaaaGcbaacciGaf83SdCMbaebaaaa@2D99@ = 0.26) yield remarkably similar fits (not shown) to the experimental data. This reveals a large insensibility to false- positive and negative noises and fluctuations in *γ *(as long as the non-stationary dense regime is avoided, Fig. 3A). The fixed (or averaged) growth rate of 52% at each round of global duplication is enough to generate networks of the size of *S. cerevisiae *starting from a few interacting "seeds" after about 20 global duplications (*i.e*. 1.52^20 ^= 4334 times more nodes with an average of one global duplication per 200 MY for 4BY). Such scenario is not *a priori *incompatible with experimental data, as we only have clear records on global duplications dating back up to 400–500 MY ago (*i.e*. only 10 to 20% of life history). Yet, these records suggest that "recent" whole genome duplications might be more frequent (every 100–150 MY) and more selective (growth rates between 10 and 25%). [Indeed, *ciona*, 16,000 genes, and *human*, ~25,000 genes, (resp. *tetraodon*, ~22,000 genes) differ by two (resp. three) whole genome duplications; this corresponds to an averaged growth rate of 25% (resp. 11%) including local duplications [49], *i.e*. (25/16)^1/2 ^= 1.25 (resp. (22/16)^1/3 ^= 1.11).] We will show, however, below, that this discrepancy is essentially resolved by redefining PPI network evolution in terms of protein binding domains instead of entire proteins. This will also provide a theoretical framework to account for both direct and indirect protein-protein interactions within multiprotein complexes.

### Direct vs indirect protein-protein interactions

The protein-protein interactions we have considered so far correspond to *direct *physical contact between *protein pairs *derived, for instance, from two-hybrid expression assays [56]. However, we expect from the proposed scale-free fit of the degree distribution (Fig. 3B) that the underlying PPI network has conserved not only pairwise interactions during evolution but also some level of network topology (see above). The emergence of locally conserved topology in PPI network evolution leads "naturally" to conserved associations or "modules" between multiple proteins [57-61] and, beyond, to recurrent "motifs" across different types of biological networks [62-71].

In fact, many biological functions are known to rely on multiple direct and indirect interactions within protein complexes. Moreover, the *combinatorial *complexity of multiple-protein interactions is likely responsible for the remarkable diversity amongst living organisms [72], despite their rather limited and largely shared genetic background (*i.e*. a few (ten) thousands genes built from a few hundreds families of homologous protein domains [18-20,73,74]).

High-throughput studies using affinity precipitation methods coupled to mass spectroscopy [75-77] have proposed some 80,000 direct and indirect protein interactions for *S. cerevisiae *(raw data) and similar data are now becoming available for several other species.

Yet, from a theoretical point of view, the evolution of *indirect *interactions is expected to depend not only on locally conserved network topology but also on the actual "combinatorial logic" between direct interactions [78,79]. This cannot be readily defined on traditional PPI network representation (*e.g*. Fig. 2) and requires a somewhat more elaborate model as we now discuss.

### Redefining PPI network evolution in terms of protein domains

Indirect protein interactions reflect the occurence of *simultaneous *direct interactions within protein complexes. This requires that some proteins have more than one binding sites to simultenaously interact with several protein partners. Indeed, proteins with a single protein-binding site can only bind to one partners at a time, underlying a simple "XOR"-like combinatorial logic. By contrast, proteins with several protein-binding sites greatly increase the combinatorial complexity of biological processes (like gene regulation or cell signaling) by adding "AND" operators to the computational logic between multiple direct interactions.

In addition, we note that binding sites are likely the primary source of asymmetric divergence in PPI network evolution, as mutations on a shared binding site will generally affect the interactions with *all *its binding partners (Fig. 2) and not just a random subset of them (Fig. S1). Hence, asymmetric divergence of binding site duplicates "naturally" results from "spontaneous symmetry breaking" due to the intrinsic evolutionary coupling of interactions sharing a common binding site. Yet, this argument of spontaneous symmetry breaking only applies to individual binding sites, not to entire proteins. Indeed, while the divergence of individual binding sites should be inherently asymmetric, this does not have to be the case *a priori *at the level of entire proteins with multiple binding sites. This is because, in principles, distinct binding sites of a protein are not necessarily coupled, thereby enabling them to evolve somewhat independently and to eventually lead, after gene duplication, to a partition of the most conserved binding site copies between each protein duplicates (*i.e*. this amounts to a "subfunctionalization" between duplicated genes, see additional file 1). Structural independence of binding sites is expected, in particular, for proteins with multiple binding sites located on different protein domains. In this case, the evolutionary symmetrization of multidomain proteins should even be further enhanced by extensive shuffling of protein domains over broad evolutionary scales [19]. Yet, we will demonstrate below that even a strong symmetrization of protein divergence at the level of protein domains, corresponding to a complete random shuffling of protein domains, is *not suffcient *to prevent the emergence of scale-free PPI networks, by constrast to predictions for symmetric models at the level of individual interactions (see discussion above and Fig. S1 in addtional file 1).

In the following, we propose to highlight this central role of protein domains in the evolution of PPI networks by simply redefining our initial asymmetric divergence model (Fig. 2) in terms of *protein-binding domains*, and assuming at first a single protein-binding site per protein-binding domain, as illustrated in Fig. 4A (see however Discussion). In particular, the normalised generating function *p*(*x*) introduced previously, Eqs.(2,3), now corresponds to the connectivity distribution of individual protein-binding domains, instead of entire proteins. This alternative representation of PPI networks provides a theoretical framework to model the evolution of the combinatorial logic underlying PPI networks, as it distinguishes mutually exclusive, direct interactions ("XOR") between protein domains (Fig. 4A, black solid lines) from cummulative, indirect interactions ("AND") within multi-protein complexes (Fig. 4A, red dashed lines).

### Combining whole genome duplication and extensive domain shuffling

As noted in the introduction, whole-genome duplications is thought to promote efficient shuffling of multi-domain proteins by enabling many accretion and deletion events of functional domains after each genome doubling. In fact, we will assume in the following that the overall shuffling of multi-domain proteins is so efficient that protein domains encoded along the genome are effectively *randomly shuffed *over long evolutionary time scales, *e.g*. > 500 MY-1 GY, as suggested by the different multi-domain combinations typically observed across distant living kingdoms [19]).

Indeed, our aim, here, is not to model the fine details of domain shuffling events on short evolutionary time scales, but instead to check the robustness of PPI network scale-free topology against the extensive shuffling of protein domains that effectively occurs over long evolutionary time scales. Assuming a random shuffling of individual protein domains implies that their evolutionary dynamics is ultimately averaged over a long series of single- and multi-domain proteins. Hence, the integrated connectivity of individual protein domains can be assumed to have evolved *independently *from their current position inside a specific single- or multi-domain protein. Besides, a more elaborate model of protein evolution detailing domain accretion and deletion events leads to virtually identical asymptotic results (not shown).

Assuming a random shuffling of independent protein domains over long evolutionary time scales is also a more stringent condition with regards to the robustness of PPI network topology against domain shuffling events. The overall topology of PPI networks is expected to be *a forceriori *less affected by actual domain shuffling events.

Finally, the assumption of random shuffling of independent protein domains is simple enough to be amenable to an exact mathematical extension of the initial model neglecting multidomain protein structures. Indeed, in the asymptotic limit, the generating function for the connectivity distribution of the global multidomain protein network, p^
 MathType@MTEF@5@5@+=feaafiart1ev1aaatCvAUfKttLearuWrP9MDH5MBPbIqV92AaeXatLxBI9gBaebbnrfifHhDYfgasaacPC6xNi=xH8viVGI8Gi=hEeeu0xXdbba9frFj0xb9qqpG0dXdb9aspeI8k8fiI+fsY=rqGqVepae9pg0db9vqaiVgFr0xfr=xfr=xc9adbaqaaeGacaGaaiaabeqaaeqabiWaaaGcbaGafmiCaaNbaKaaaaa@2D4C@(*x*), can be derived *a posteriori *by reconstructing multidomain proteins from a poissonian linking of successive protein domains whose connectivies are characterized by the generating function *p*(*x*) = ∑_*k*≥1_*p*_*k*_*x*^*k *^and randomly distributed along the genome. Hence, *p*_*k *_is the probability to find a protein domain with connectivity *k *at a given location along the genome. We introduce a new parameter *λ*, corresponding to the probability to form a covalent connection between successive protein-binding domains encoded along the genome. Then, the respective contributions of single, double, triple domain proteins to the overall multidomain generating function p^
 MathType@MTEF@5@5@+=feaafiart1ev1aaatCvAUfKttLearuWrP9MDH5MBPbIqV92AaeXatLxBI9gBaebbnrfifHhDYfgasaacPC6xNi=xH8viVGI8Gi=hEeeu0xXdbba9frFj0xb9qqpG0dXdb9aspeI8k8fiI+fsY=rqGqVepae9pg0db9vqaiVgFr0xfr=xfr=xc9adbaqaaeGacaGaaiaabeqaaeqabiWaaaGcbaGafmiCaaNbaKaaaaa@2D4C@(*x*) become, *p*(*x*)(1 - *λ*), *p*(*x*)*λp*(*x*)(1 - *λ*), *p*(*x*)*λp*(*x*)*λp*(*x*)(1 - *λ*), etc, to account for the probability to find a given multidomain protein whose global connectivity is summed over its individual domains, *e.g*., *p*_*k*_*λp*_*k*'_(1 - *λ*), with global connectivity *k *+ *k'*.

Hence, summing over all possible multidomain proteins finally yields for the overall generating function p^
 MathType@MTEF@5@5@+=feaafiart1ev1aaatCvAUfKttLearuWrP9MDH5MBPbIqV92AaeXatLxBI9gBaebbnrfifHhDYfgasaacPC6xNi=xH8viVGI8Gi=hEeeu0xXdbba9frFj0xb9qqpG0dXdb9aspeI8k8fiI+fsY=rqGqVepae9pg0db9vqaiVgFr0xfr=xfr=xc9adbaqaaeGacaGaaiaabeqaaeqabiWaaaGcbaGafmiCaaNbaKaaaaa@2D4C@(*x*) = *p*(*x*)(1 + *λp*(*x*) + *λ*^2^*p*^2^(*x*) + ...)(1 - *λ*),

p^(x)=(1−λ)p(x)1−λp(x)=1−1−p(x)1−λp(x)
 MathType@MTEF@5@5@+=feaafiart1ev1aaatCvAUfKttLearuWrP9MDH5MBPbIqV92AaeXatLxBI9gBaebbnrfifHhDYfgasaacPC6xNi=xI8qiVKYPFjYdHaVhbbf9v8qqaqFr0xc9vqFj0dXdbba91qpepeI8k8fiI+fsY=rqGqVepae9pg0db9vqaiVgFr0xfr=xfr=xc9adbaqaaeGacaGaaiaabeqaaeqabiWaaaGcbaGafmiCaaNbaKaacqGGOaakcqWG4baEcqGGPaqkcqGH9aqpjuaGdaWcaaqaaiabcIcaOiabigdaXiabgkHiTGGaciab=T7aSjabcMcaPiabdchaWjabcIcaOiabdIha4jabcMcaPaqaaiabigdaXiabgkHiTiab=T7aSjabdchaWjabcIcaOiabdIha4jabcMcaPaaakiabg2da9iabigdaXiabgkHiTKqbaoaalaaabaGaeGymaeJaeyOeI0IaemiCaaNaeiikaGIaemiEaGNaeiykaKcabaGaeGymaeJaeyOeI0Iae83UdWMaemiCaaNaeiikaGIaemiEaGNaeiykaKcaaaaa@5683@

Although non-protein-binding domains are omitted here for simplicity, they can readily be taken into account by including a fraction of disconnected, non-protein-binding domains in *p*(*x*). Eq. (5) implies, in particular, an exponential distribution of multi-domain proteins, in agreement with actual distributions [80,81], with an average of 1/(1 - *λ*) protein-binding sites per protein. While *p*(*x*) now reflects the independent evolution of single protein-binding domains, Eq. (5) shows that it also controls the asymptotic properties of the derived multi-domain networks p^
 MathType@MTEF@5@5@+=feaafiart1ev1aaatCvAUfKttLearuWrP9MDH5MBPbIqV92AaeXatLxBI9gBaebbnrfifHhDYfgasaacPC6xNi=xH8viVGI8Gi=hEeeu0xXdbba9frFj0xb9qqpG0dXdb9aspeI8k8fiI+fsY=rqGqVepae9pg0db9vqaiVgFr0xfr=xfr=xc9adbaqaaeGacaGaaiaabeqaaeqabiWaaaGcbaGafmiCaaNbaKaaaaa@2D4C@(*x*); in particular, for biologically relevant cases with Γ_o _> 1 > Γ_n_, we obtain from Eq. (3) the following asymptotic expansion in the vicinity of *x *= 1,

p^(x)~1−…−Aα1−λ(1−x)α−…
 MathType@MTEF@5@5@+=feaafiart1ev1aaatCvAUfKttLearuWrP9MDH5MBPbIqV92AaeXatLxBI9gBaebbnrfifHhDYfgasaacPC6xNi=xI8qiVKYPFjYdHaVhbbf9v8qqaqFr0xc9vqFj0dXdbba91qpepeI8k8fiI+fsY=rqGqVepae9pg0db9vqaiVgFr0xfr=xfr=xc9adbaqaaeGacaGaaiaabeqaaeqabiWaaaGcbaGafmiCaaNbaKaacqGGOaakcqWG4baEcqGGPaqkcqGG+bGFcqaIXaqmcqGHsislcqWIMaYscqGHsisldaWcaaqaaiabdgeabnaaBaaaleaaiiGacqWFXoqyaeqaaaGcbaGaeGymaeJaeyOeI0Iae83UdWgaaiabcIcaOiabigdaXiabgkHiTiabdIha4jabcMcaPmaaCaaaleqabaGae8xSdegaaOGaeyOeI0IaeSOjGSeaaa@45A0@

which implies that degree distributions of multi-domain protein networks p^
 MathType@MTEF@5@5@+=feaafiart1ev1aaatCvAUfKttLearuWrP9MDH5MBPbIqV92AaeXatLxBI9gBaebbnrfifHhDYfgasaacPC6xNi=xH8viVGI8Gi=hEeeu0xXdbba9frFj0xb9qqpG0dXdb9aspeI8k8fiI+fsY=rqGqVepae9pg0db9vqaiVgFr0xfr=xfr=xc9adbaqaaeGacaGaaiaabeqaaeqabiWaaaGcbaGafmiCaaNbaKaaaaa@2D4C@_*k *_increase with respect to the underlying single-domain interaction network *p*_*k *_as p^
 MathType@MTEF@5@5@+=feaafiart1ev1aaatCvAUfKttLearuWrP9MDH5MBPbIqV92AaeXatLxBI9gBaebbnrfifHhDYfgasaacPC6xNi=xH8viVGI8Gi=hEeeu0xXdbba9frFj0xb9qqpG0dXdb9aspeI8k8fiI+fsY=rqGqVepae9pg0db9vqaiVgFr0xfr=xfr=xc9adbaqaaeGacaGaaiaabeqaaeqabiWaaaGcbaGafmiCaaNbaKaaaaa@2D4C@_*k *_~ *p*_*k*_/(1 - *λ*) for large *k*, while the fraction of proteins with a single binding partner p^
 MathType@MTEF@5@5@+=feaafiart1ev1aaatCvAUfKttLearuWrP9MDH5MBPbIqV92AaeXatLxBI9gBaebbnrfifHhDYfgasaacPC6xNi=xH8viVGI8Gi=hEeeu0xXdbba9frFj0xb9qqpG0dXdb9aspeI8k8fiI+fsY=rqGqVepae9pg0db9vqaiVgFr0xfr=xfr=xc9adbaqaaeGacaGaaiaabeqaaeqabiWaaaGcbaGafmiCaaNbaKaaaaa@2D4C@_1 _decreases at the same time as p^
 MathType@MTEF@5@5@+=feaafiart1ev1aaatCvAUfKttLearuWrP9MDH5MBPbIqV92AaeXatLxBI9gBaebbnrfifHhDYfgasaacPC6xNi=xH8viVGI8Gi=hEeeu0xXdbba9frFj0xb9qqpG0dXdb9aspeI8k8fiI+fsY=rqGqVepae9pg0db9vqaiVgFr0xfr=xfr=xc9adbaqaaeGacaGaaiaabeqaaeqabiWaaaGcbaGafmiCaaNbaKaaaaa@2D4C@_1 _= p^
 MathType@MTEF@5@5@+=feaafiart1ev1aaatCvAUfKttLearuWrP9MDH5MBPbIqV92AaeXatLxBI9gBaebbnrfifHhDYfgasaacPC6xNi=xH8viVGI8Gi=hEeeu0xXdbba9frFj0xb9qqpG0dXdb9aspeI8k8fiI+fsY=rqGqVepae9pg0db9vqaiVgFr0xfr=xfr=xc9adbaqaaeGacaGaaiaabeqaaeqabiWaaaGcbaGafmiCaaNbaKaaaaa@2D4C@'(0) = (1 - *λ*)*p'*(0) = (1 - *λ*)*p*_1 _(see Fig. 4B). From a biological perspective, note that the scale-free degree distribution of such multi-domain protein networks results from an asymmetric divergence of individual binding sites (or domains) rather than an asymmetric divergence of global protein architectures. This has also biological consequences for the functionalization of duplicated genes (see additional file ). In particular, random (symmetric) "subfunctionalization" between protein duplicates at the level of protein domains does *not *prevent the emergence of scale-free networks with locally conserved topology, by contrast to random link "complementation" at the level of individual interactions (Fig. S1) which leads to exponential networks without conserved topology (see Supporting Information).

Hence, domain shuffling of multi-domain proteins provides a powerful, yet non-disruptive source of combinatorial innovation, as it preserves essential topological features inherited from the underlying protein-domain interaction network evolution.

Finally, comparison with experimental data sets including indirect protein-protein interactions [75-77] is made by adopting a statistical implementation of the "combinatorial logic" discussed above (see Supporting Information). It is based on a Dijkstra algorithm that estimates the relative importance of all possible indirect interactions between multi-domain (and single-domain) proteins for each PPI network realization. Figs. 4B &4C show rather good fits of experimental data sets corresponding to an estimated 30% to 60% coverage of actual PPI networks [75-77] (see, however, Supporting Information). The two adjusted parameters, *γ *= 0.1 and *λ *= 0.3, correspond to a network growth rate of 20% (*i.e*. 1 + 2*γ*) and an average of 1.5 (*i.e*. 1/(1 - *λ*)) protein-binding sites (domains) per protein in agreement with broad estimates for these biological parameters (see above and [80,81]). This also confirms that the properties of PPI networks we have predicted from first principles (*i.e. i) *exponential dynamics and *ii) *symmetry breaking) are already transparent from partial data sets.

## Discussion

In this paper, we establish the statistical consequences of successive whole genome duplications and divergence asymmetry between gene duplicates on both *i) *evolutionary conservation and *ii) *emerging topological properties of PPI networks. The evolutionary dynamics of non-conserved networks implies that all evolutionary traces are erased exponentially fast from the network and its underlying genome over typical WGD time scales (*e.g*. 100 MY). Hence, evolutionary conserved networks are presumably the only biologically relevant PPI networks that may arise through whole genome duplications. We have also demonstrated that they necessarily present a scale-free topology that is robust to extensive domain shuffling of their multiple domain proteins.

Other evolutionary processes than WGD and domain shuffling have not been included in the main text above, for simplicity. Yet, additional PPI network features can also be taken into account. We have investigated, in particular, the roles of 3 additional well-documented features of PPI network evolution, which we discuss below. They are *i) *protein homo-oligomerization, *ii) *protein domains with multiple binding sites and, finally, *iii) *other duplication-divergence events at smaller genomic scale than entire genome (*i.e*. from single gene to partial genome duplication). Yet, we have found that none of these additional PPI network features significantly affect the general conclusions of the present study.

### • i) Protein homo-oligomerization

The possibility of protein homo-oligomerization can be explicitly taken into account by introducing 2 types of nodes corresponding respectively to *i) *self-interacting proteins with self-link loops and *ii) *non-self-interacting proteins without self-link loops, see Fig. S2 and Supporting Information. Available data on PPI networks reveals that about 10 to 15% of interacting proteins are self-interacting [38,39]. Empiral evidence have also been reported on the higher overall connectivity and interconnectivity of homodimer proteins in PPI networks [82]. In principle, the detailed evolution of PPI network conservation and topology is affected by self-link loops which provide a source of duplication-derived *de novo *interactions between "old" and "new" copies of duplicated self-interacting proteins, Fig. S2. However, the general conservation and topological properties of PPI networks turn out to be little affected by the presence of self-link loops, in the asymptotic limits of large PPI networks and large node degrees (see Supporting Information for detailed proof). In a nutshell, this is because conservation and topology of PPI networks are controlled by the exponential increase of their node degrees while the contribution of *de novo *interactions arising from duplicated self-interacting proteins can at most lead to a linear increase of node degrees, with a maximum increment of +1 link per duplication event and protein. Thus, although an abundance of self-interacting proteins would significantly affect the evolution of low connectivity proteins, it could *not *lead to a change of topological regimes for the highly connected nodes of the PPI networks (*e.g*. from exponential to scale-free node degree distribution or vice versa). Hence, to a first approximation, self-interacting proteins can be simply ignored to establish the asymptotic conservation and topology regimes of PPI network evolution, as we have done in the main text and Fig. 3A. Note, however, that the actual power law exponents of scale-free node degree distributions might nonetheless be affected by *de novo *interactions arising from duplicated self-interacting proteins (see Supporting Information for details). In addition, self-link loops might also be important for the evolution of certain network motifs whose initial emergence might precisely depend on the presence of self-interacting proteins (*e.g*. the triangle motif unless one triangle at least is already present in the initial network).

### • ii) Protein domains with multiple binding sites

The possibility of having protein interfaces involving more than two proteins at a time (*e.g*., the hetero-trimeric fibrinogen) is not currently included in the model. Actually, the average number of binding sites per protein-binding domains is around 1.3, with about 80% of protein-binding domains having a single binding site [83] (except for self-interacting domains forming homo-oligomeric self-assemblies, which require, as expected, at least 2 binding sites, see table 2 in [83].) Yet, in principle, the evolution of protein-binding domains with multiple binding sites can be taken effectively into account, at least numerically, by introducing a strong physical correlation between successive single-binding-site "domains". However, we want to stress that our main results regarding protein-binding domains do not concern nor rely on the detailed evolutionary correlation of binding sites and domain shuffling mechanisms. Indeed, by assuming only single-binding-site domains, we have demonstrated that even the most extensive shuffling of binding site/domain orders, implying the loss of all correlation along the primary sequence, does not qualitatively affect the general conservation and topological properties of emerging PPI networks under whole genome duplications. Hence, it is quite clear and confirmed by simulations (not shown) that introducing physical correlation between successive binding sites/domains has *a forceriori *even less effect on the general evolutionary regimes, we have predicted above.

### • iii) Duplication-divergence events at smaller genomic scales

Finally, beyond whole genome duplication, duplication-divergence events are also known to occur at smaller genomic scales from single gene to partial genome duplication. Moreover, local duplications/deletions may also lead to exponential dynamics of PPI network evolution if they are selected independently in parallel. A general model for PPI network evolution under duplication-divergence processes at *any *genomic scale (from single gene to whole genome) and allowing also for *variations *in all evolutionary parameters {γi(n)}
 MathType@MTEF@5@5@+=feaafiart1ev1aaatCvAUfKttLearuWrP9MDH5MBPbIqV92AaeXatLxBI9gBaebbnrfifHhDYfgasaacPC6xNi=xH8viVGI8Gi=hEeeu0xXdbba9frFj0xb9qqpG0dXdb9aspeI8k8fiI+fsY=rqGqVepae9pg0db9vqaiVgFr0xfr=xfr=xc9adbaqaaeGacaGaaiaabeqaaeqabiWaaaGcbaGaei4EaShcciGae83SdC2aa0baaSqaaiabbMgaPbqaaiabcIcaOiabd6gaUjabcMcaPaaakiabc2ha9baa@3528@ over evolutionary time scale *n *is presented in ref [47]. It confirms and generalizes the conclusions of the present study focussing on whole genome duplications.

Interestingly, recent evolutionary records (< 500 MY) for specific eukaryotes from various kingdoms, *e.g*. [23,33], suggest that whole genome duplications have been a significant factor in the overall expansion of ancestral genomes [23,33], while local duplications have been mainly responsible for the expansion of specific gene families. It will be interesting to see whether this is a general trend or not as new complete eukaryote sequences will become available.

This difference in typical selection pattern of gene duplicates from either whole genome or local duplications may possibly reflect their opposite dosage effects on cellular activity and ultimately correspond to two evolutionary paradigms reminiscent to Monod's "chance and necessity" principles [84]. Indeed, random local duplications of essential genes are thought to be generally detrimental by the dosage imbalance they initially induce, thereby raising the odds for their rapid nonfunctionalization [85-87], unless they specifically happen to be beneficial under concomitant environmental changes [51]. Hence, the typical fate of random local duplications might be primarily driven by immediate "necessity" rather than "chance" and eventually lead to the expansion of specific gene families through series of beneficial local duplications. By contrast, rapid nonfunctionalization of duplicates following a whole genome duplication should be typically opposed by dosage effect, in particular, for highly expressed genes and for genes involved in multiprotein complexes or metabolic pathways [33]. This is because whole genome duplications initially preserve correct relative dosage between expressed genes, while subsequent random nonfunctionalizations disrupt this initial dosage balance.

Preventing rapid asymmetric divergence between duplicates from recent whole genome duplications appears, in the end, to increase their chance of neo- or subfunctionalization by favoring longer genetic drift rather than early functional loss. Hence, by contrast with local duplications, the typical fate of gene duplicates under whole genome duplication might be largely driven by (long term) chance rather than (immediate) necessity. It is also reflected in the random pattern of reciprocal gene loss associated with multiple speciation events that typically follow a whole genome duplication [13,21]. This prevalence of chance over necessity following whole genome duplications further supports the stochastic and statistical framework we have adopted here to model the evolution of PPI networks under whole genome duplication.

## Conclusion

In this paper, we argue that, large scale topological features of PPI networks emerge spontaneously in the course of evolution under simple duplication/deletion events [45], regardless of the specific evolutionary advantages individual proteins might have been selected for. While other selection drives than mere protein domain conservation might have also played a role, they do not appear to have been necessary nor prevailing factors to shape the large scale topology of PPI networks. For instance, the repulsion of protein hubs into largely independent network modules (*i.e*. the so-called network "disassortativity" property [42,50]) is predicted here (Figs. 3C &4B) without any specific selection pressure being ever invoked in favor of such network motifs. Yet, we showed that the exponential dynamics of PPI network evolution under genome duplication *requires *an asymmetric divergence of protein duplicates. Such asymmetric divergence arises, however, "naturally" at the level of protein-binding sites or domains (through "spontaneous symmetry breaking") and is robust to extensive domain shuffling of multi-domain proteins.

From a more general perspective, the context of accelerating genome sequencing projects calls for a broader and inevitably more statistical understanding of biological network evolution, beyond the accumulation of details for particular evolutionary transitions of specific species. The analysis of PPI networks over broad evolutionary scales can only be based on a few well-established evolutionary mechanisms shared across a wide variety of organisms. As novel whole genome duplications are now routinely discovered in newly sequenced eukaryote genomes, *e.g*. [23,33], it is clear that these rare but dramatic simultaneous changes in genome content must have had a major impact on the long time scale evolution of eukaryote genomes and, hence, resulting biological networks. This study demonstrates the expected biological implications of such successive genome duplications in terms of both conservation and topology of PPI networks. In particular, it shows from first principles, that scale-free topologies of PPI networks are a simple consequence of their evolutionary conservation. It also highlights the importance and origin of the divergence asymmetry between gene duplicates, as well as the overall robustness of the resulting scale-free topology to domain shuffling of multi-domain proteins.

## Method

### Mathematical solution of the model

Our formal approach is based on the use of generating functions to capture the statistical properties of emerging PPI networks under WGD. In particular, the generating function of the average number of protein nodes ⟨Nk(n)
 MathType@MTEF@5@5@+=feaafiart1ev1aaatCvAUfKttLearuWrP9MDH5MBPbIqV92AaeXatLxBI9gBaebbnrfifHhDYfgasaacPC6xNi=xH8viVGI8Gi=hEeeu0xXdbba9frFj0xb9qqpG0dXdb9aspeI8k8fiI+fsY=rqGqVepae9pg0db9vqaiVgFr0xfr=xfr=xc9adbaqaaeGacaGaaiaabeqaaeqabiWaaaGcbaGaemOta40aa0baaSqaaiabdUgaRbqaaiabcIcaOiabd6gaUjabcMcaPaaaaaa@319B@⟩ with *k *binding partners after *n *WGD steps is defined as,

F(n)(x)=∑k≥0〈Nk(n)〉xk
 MathType@MTEF@5@5@+=feaafiart1ev1aaatCvAUfKttLearuWrP9MDH5MBPbIqV92AaeXatLxBI9gBaebbnrfifHhDYfgasaacPC6xNi=xI8qiVKYPFjYdHaVhbbf9v8qqaqFr0xc9vqFj0dXdbba91qpepeI8k8fiI+fsY=rqGqVepae9pg0db9vqaiVgFr0xfr=xfr=xc9adbaqaaeGacaGaaiaabeqaaeqabiWaaaGcbaGaemOray0aaWbaaSqabeaacqGGOaakcqWGUbGBcqGGPaqkaaGccqGGOaakcqWG4baEcqGGPaqkcqGH9aqpdaaeqbqaaiabgMYiHlabd6eaonaaDaaaleaacqWGRbWAaeaacqGGOaakcqWGUbGBcqGGPaqkaaGccqGHQms8cqWG4baEdaahaaWcbeqaaiabdUgaRbaaaeaacqWGRbWAcqGHLjYScqaIWaamaeqaniabggHiLdaaaa@473A@

As discussed in Results, a general model for PPI network evolution under WGD allows for an asymmetric divergence of duplicated genes, Fig. 2. Hence, each WGD step (*n*) → (*n *+ 1) corresponds to the following functional recurrence between consecutive generating functions *F*^(*n*) ^and *F*^(*n *+ 1)^,

*F*^(*n*+1)^(*x*) = *F*^(*n*)^(*A*_n_(*x*)) + *F*^(*n*)^(*A*_o_(*x*))

where *A*_i_(*x*) = (*γx *+ *δ*)(*γ*_i_*x *+ *δ*_i_), for i = n, o and, *γ*, *γ*_n _and *γ*_o _[resp. *δ*, *δ*_n _and *δ*_o_] correspond to the probabilities to preserve [resp. delete] the duplication-derived interactions between "old" and "new" duplicated nodes, as depicted in Fig. 2. The functional recurrence Eq. 8 is derived as follows. Since each node is initially duplicated, *F*^(*n*+1)^(*x*), which essentially counts the number of nodes according to their degree *k *≥ 0, is the sum of two *F*^(*n*)^(*x*) corresponding, respectively, to the "old" and "new" nodes in the duplicated network. The variable *x *in *F*^(*n*)^(*x*), whose successive powers *x*^*k *^essentially count the number of links (*k*) around each node of degree *k*, should then be replaced by *x*^2 ^(since each node degree can at most double) and eventually be substituted as *x *ⅾ *γ*_*i*_*x *+ *δ*_*i*_, where *γ*_*i *_[resp. *δ*_*i *_= 1 - *γ*_*i*_] corresponds to the probability to keep [resp. delete] each link emerging from each node of the duplicated graph. Hence, at each WGD step (*n*) ⅾ (*n *+ 1), the generating function recurrence for PPI network evolution with asymmetric divergence of duplicated proteins becomes Eq. 8 (see Supporting Information for proof details).

Note, that there are two types of time scales in this model of PPI network evolution: one which is slow corresponds to the long time decay of ancestral interactions between "old" genes, while the other one is faster (*e.g*. 10–100 MY) and corresponds to the spontaneous symmetry breaking between "old" and "new" duplicate copies and the concommitant deletion of many "new" duplicates. In particular, we do not introduce distinct time scales for spontaneous symmetry breaking and deletion of "new" genes, since these two steps are not assumed to be distinct phenomena but rather simultaneous processes that cannot be formally decoupled.

The overall graph dynamics through successive global duplications is clearly exponential as anticipated; in particular, the total number of nodes grows as *F*^(*n*)^(1) = *A*·2^*n*^, where *A *is the initial number of nodes, and the number of links scales as ⟨*L*^(*n*)^⟩ ∝ (2*γ *+ *γ*_o _+ *γ*_n_)^*n*^. We remove permanently disconnected nodes from the list of relevant nodes, assuming that they correspond to proteins that have in fact lost their function and are eventually eliminated from the genome. To this end, we redefine the graph size as, 〈N(n)〉=∑k≥1〈Nk(n)〉
 MathType@MTEF@5@5@+=feaafiart1ev1aaatCvAUfKttLearuWrP9MDH5MBPbIqV92AaeXatLxBI9gBaebbnrfifHhDYfgasaacPC6xNi=xH8viVGI8Gi=hEeeu0xXdbba9frFj0xb9qqpG0dXdb9aspeI8k8fiI+fsY=rqGqVepae9pg0db9vqaiVgFr0xfr=xfr=xc9adbaqaaeGacaGaaiaabeqaaeqabiWaaaGcbaGaeyykJeUaemOta40aaWbaaSqabeaacqGGOaakcqWGUbGBcqGGPaqkaaGccqGHQms8cqGH9aqpdaaeqaqaaiabgMYiHlabd6eaonaaDaaaleaacqWGRbWAaeaacqGGOaakcqWGUbGBcqGGPaqkaaGccqGHQms8aSqaaiabdUgaRjabgwMiZkabigdaXaqab0GaeyyeIuoaaaa@441C@, where 〈N0(n)〉
 MathType@MTEF@5@5@+=feaafiart1ev1aaatCvAUfKttLearuWrP9MDH5MBPbIqV92AaeXatLxBI9gBaebbnrfifHhDYfgasaacPC6xNi=xH8viVGI8Gi=hEeeu0xXdbba9frFj0xb9qqpG0dXdb9aspeI8k8fiI+fsY=rqGqVepae9pg0db9vqaiVgFr0xfr=xfr=xc9adbaqaaeGacaGaaiaabeqaaeqabiWaaaGcbaGaeyykJeUaemOta40aa0baaSqaaiabicdaWaqaaiabcIcaOiabd6gaUjabcMcaPaaakiabgQYiXdaa@34B7@ has been removed, and introduce a normalized generating function *p*^(*n*)^(*x*) for the mean degree distribution,

p(n)(x)=∑k≥1pk(n)xk,wherepk(n)=〈Nk(n)〉〈N(n)〉
 MathType@MTEF@5@5@+=feaafiart1ev1aaatCvAUfKttLearuWrP9MDH5MBPbIqV92AaeXatLxBI9gBaebbnrfifHhDYfgasaacPC6xNi=xI8qiVKYPFjYdHaVhbbf9v8qqaqFr0xc9vqFj0dXdbba91qpepeI8k8fiI+fsY=rqGqVepae9pg0db9vqaiVgFr0xfr=xfr=xc9adbaqaaeGacaGaaiaabeqaaeqabiWaaaGcbaqbaeqabeWaaaqaaiabdchaWnaaCaaaleqabaGaeiikaGIaemOBa4MaeiykaKcaaOGaeiikaGIaemiEaGNaeiykaKIaeyypa0ZaaabuaeaacqWGWbaCdaqhaaWcbaGaem4AaSgabaGaeiikaGIaemOBa4MaeiykaKcaaOGaemiEaG3aaWbaaSqabeaacqWGRbWAaaaabaGaem4AaSMaeyyzImRaeGymaedabeqdcqGHris5aOGaeiilaWcabaGaee4DaCNaeeiAaGMaeeyzauMaeeOCaiNaeeyzaugabaGaemiCaa3aa0baaSqaaiabdUgaRbqaaiabcIcaOiabd6gaUjabcMcaPaaakiabg2da9KqbaoaalaaabaGaeyykJeUaemOta40aa0baaeaacqWGRbWAaeaacqGGOaakcqWGUbGBcqGGPaqkaaGaeyOkJepabaGaeyykJeUaemOta40aaWbaaeqabaGaeiikaGIaemOBa4MaeiykaKcaaiabgQYiXdaaaaaaaa@64FE@

Absolute and relative generating functions are related through,

F(n)(x)=p(n)(x)〈N(n)〉+〈N0(n)〉
 MathType@MTEF@5@5@+=feaafiart1ev1aaatCvAUfKttLearuWrP9MDH5MBPbIqV92AaeXatLxBI9gBaebbnrfifHhDYfgasaacPC6xNi=xI8qiVKYPFjYdHaVhbbf9v8qqaqFr0xc9vqFj0dXdbba91qpepeI8k8fiI+fsY=rqGqVepae9pg0db9vqaiVgFr0xfr=xfr=xc9adbaqaaeGacaGaaiaabeqaaeqabiWaaaGcbaGaemOray0aaWbaaSqabeaacqGGOaakcqWGUbGBcqGGPaqkaaGccqGGOaakcqWG4baEcqGGPaqkcqGH9aqpcqWGWbaCdaahaaWcbeqaaiabcIcaOiabd6gaUjabcMcaPaaakiabcIcaOiabdIha4jabcMcaPiabgMYiHlabd6eaonaaCaaaleqabaGaeiikaGIaemOBa4MaeiykaKcaaOGaeyOkJeVaey4kaSIaeyykJeUaemOta40aa0baaSqaaiabicdaWaqaaiabcIcaOiabd6gaUjabcMcaPaaakiabgQYiXdaa@4E53@

Inserting this expression (10) in recurrence (8) gives a closed relation between successive p˜
 MathType@MTEF@5@5@+=feaafiart1ev1aaatCvAUfKttLearuWrP9MDH5MBPbIqV92AaeXatLxBI9gBaebbnrfifHhDYfgasaacPC6xNi=xH8viVGI8Gi=hEeeu0xXdbba9frFj0xb9qqpG0dXdb9aspeI8k8fiI+fsY=rqGqVepae9pg0db9vqaiVgFr0xfr=xfr=xc9adbaqaaeGacaGaaiaabeqaaeqabiWaaaGcbaGafmiCaaNbaGaaaaa@2D4B@^(*n*)^(*x*) = *p*^(*n*)^(*x*) - 1,

p˜(n+1)(x)=p˜(n)(An(x))+p˜(n)(Ao(x))Δ(n)
 MathType@MTEF@5@5@+=feaafiart1ev1aaatCvAUfKttLearuWrP9MDH5MBPbIqV92AaeXatLxBI9gBaebbnrfifHhDYfgasaacPC6xNi=xI8qiVKYPFjYdHaVhbbf9v8qqaqFr0xc9vqFj0dXdbba91qpepeI8k8fiI+fsY=rqGqVepae9pg0db9vqaiVgFr0xfr=xfr=xc9adbaqaaeGacaGaaiaabeqaaeqabiWaaaGcbaGafmiCaaNbaGaadaahaaWcbeqaaiabcIcaOiabd6gaUjabgUcaRiabigdaXiabcMcaPaaakiabcIcaOiabdIha4jabcMcaPiabg2da9maalaaabaGafmiCaaNbaGaadaahaaWcbeqaaiabcIcaOiabd6gaUjabcMcaPaaakiabcIcaOiabdgeabnaaBaaaleaacqqGUbGBaeqaaOGaeiikaGIaemiEaGNaeiykaKIaeiykaKIaey4kaSIafmiCaaNbaGaadaahaaWcbeqaaiabcIcaOiabd6gaUjabcMcaPaaakiabcIcaOiabdgeabnaaBaaaleaacqqGVbWBaeqaaOGaeiikaGIaemiEaGNaeiykaKIaeiykaKcabaGaeuiLdq0aaWbaaSqabeaacqGGOaakcqWGUbGBcqGGPaqkaaaaaaaa@5516@

where Δ^(*n*) ^is the ratio between consecutive numbers of connected nodes,

Δ^(*n*) ^= ⟨*N*^(*n*+1)^⟩/⟨*N*^(*n*)^⟩ = 2 - *p*^(*n*)^(*δδ*_n_) - *p*(*n*)(*δδ*_o_) ≤ 2. The evolution of the mean degree is obtained by taking the first derivative of (11) at *x *= 1,

∂xp(n+1)(1)=Γn+ΓoΔ(n)∂xp(n)(1)
 MathType@MTEF@5@5@+=feaafiart1ev1aaatCvAUfKttLearuWrP9MDH5MBPbIqV92AaeXatLxBI9gBaebbnrfifHhDYfgasaacPC6xNi=xI8qiVKYPFjYdHaVhbbf9v8qqaqFr0xc9vqFj0dXdbba91qpepeI8k8fiI+fsY=rqGqVepae9pg0db9vqaiVgFr0xfr=xfr=xc9adbaqaaeGacaGaaiaabeqaaeqabiWaaaGcbaGaeyOaIy7aaSbaaSqaaiabdIha4bqabaGccqWGWbaCdaahaaWcbeqaaiabcIcaOiabd6gaUjabgUcaRiabigdaXiabcMcaPaaakiabcIcaOiabigdaXiabcMcaPiabg2da9KqbaoaalaaabaGaeu4KdC0aaSbaaeaacqqGUbGBaeqaaiabgUcaRiabfo5ahnaaBaaabaGaee4Ba8gabeaaaeaacqqHuoardaahaaqabeaacqGGOaakcqWGUbGBcqGGPaqkaaaaaOGaeyOaIy7aaSbaaSqaaiabdIha4bqabaGccqWGWbaCdaahaaWcbeqaaiabcIcaOiabd6gaUjabcMcaPaaakiabcIcaOiabigdaXiabcMcaPaaa@4FD8@

where Γ_n _= *γ *+ *γ*_n _= A′n(1)
 MathType@MTEF@5@5@+=feaafiart1ev1aaatCvAUfKttLearuWrP9MDH5MBPbIqV92AaeXatLxBI9gBaebbnrfifHhDYfgasaacPC6xNi=xH8viVGI8Gi=hEeeu0xXdbba9frFj0xb9qqpG0dXdb9aspeI8k8fiI+fsY=rqGqVepae9pg0db9vqaiVgFr0xfr=xfr=xc9adbaqaaeGacaGaaiaabeqaaeqabiWaaaGcbaGafmyqaeKbauaadaWgaaWcbaGaeeOBa4gabeaakiabcIcaOiabigdaXiabcMcaPaaa@3125@ and Γ_o _= *γ *+ *γ*_o _= A′o(1)
 MathType@MTEF@5@5@+=feaafiart1ev1aaatCvAUfKttLearuWrP9MDH5MBPbIqV92AaeXatLxBI9gBaebbnrfifHhDYfgasaacPC6xNi=xH8viVGI8Gi=hEeeu0xXdbba9frFj0xb9qqpG0dXdb9aspeI8k8fiI+fsY=rqGqVepae9pg0db9vqaiVgFr0xfr=xfr=xc9adbaqaaeGacaGaaiaabeqaaeqabiWaaaGcbaGafmyqaeKbauaadaWgaaWcbaGaee4Ba8gabeaakiabcIcaOiabigdaXiabcMcaPaaa@3127@ hereafter. For each type of node i = n, o, Γ_i _corresponds to the average rate of connectivity change between WGDs, *k *→ *k*Γ_i_. Hence, in particular, the connectivity of the most conserved duplicates decrease or increase as kΓom
 MathType@MTEF@5@5@+=feaafiart1ev1aaatCvAUfKttLearuWrP9MDH5MBPbIqV92AaeXatLxBI9gBaebbnrfifHhDYfgasaacPC6xNi=xH8viVGI8Gi=hEeeu0xXdbba9frFj0xb9qqpG0dXdb9aspeI8k8fiI+fsY=rqGqVepae9pg0db9vqaiVgFr0xfr=xfr=xc9adbaqaaeGacaGaaiaabeqaaeqabiWaaaGcbaGaem4AaSMaeu4KdC0aa0baaSqaaiabb+gaVbqaaiabd2gaTbaaaaa@318F@ under *m *successive WGDs: the case Γ_o _< 1 corresponds to an exponential decrease of connectivity and eventual disappearence of any given node of the network. By contrast, the case Γ_o _> 1 corresponds to a connectivity increase of the "old" duplicate descents and, hence, to an overall conservation of the PPI network in the course of evolution under WGDs. We will now show that the same criteria on Γ_o _governs not only the evolutionary conservation but also the topology of the emerging PPI networks under WGDs.

We will limit the discussion here to degree distributions approaching a stationary regimes *p*^(*n*)^(*x*) → *p*(*x*) with a *finite *mean degree 1 ≤ *p*'(1) < ∞. This seems to cover the most biologically relevant networks; for completeness, other cases are discussed elsewhere [47]. From (12) and the condition of finite mean degree, we readily obtain that Δ^(*n*) ^→ Γ_n _+ Γ_o _≤ 2, which implies that the network evolution is asymptotically equivalent in terms of connected nodes and links,

⟨*N*^(*n*+1)^⟩/⟨*N*^(*n*)^⟩ → ⟨*L*^(*n*+1)^⟩/⟨*L*^(*n*)^⟩ = Γ_n _+ Γ_o_

This condition can be shown [47] to ensure that the evolution of the *ensemble average *of networks (Eq. 7) indeed reflects the "typical" evolution of PPI networks under global duplication.

The stationary degree distribution is then solution of the functional equation, with p˜
 MathType@MTEF@5@5@+=feaafiart1ev1aaatCvAUfKttLearuWrP9MDH5MBPbIqV92AaeXatLxBI9gBaebbnrfifHhDYfgasaacPC6xNi=xH8viVGI8Gi=hEeeu0xXdbba9frFj0xb9qqpG0dXdb9aspeI8k8fiI+fsY=rqGqVepae9pg0db9vqaiVgFr0xfr=xfr=xc9adbaqaaeGacaGaaiaabeqaaeqabiWaaaGcbaGafmiCaaNbaGaaaaa@2D4B@(*x*) = *p*(*x*) - 1,

p˜(x)=p˜(An(x))+p˜(Ao(x))Γn+Γo
 MathType@MTEF@5@5@+=feaafiart1ev1aaatCvAUfKttLearuWrP9MDH5MBPbIqV92AaeXatLxBI9gBaebbnrfifHhDYfgasaacPC6xNi=xI8qiVKYPFjYdHaVhbbf9v8qqaqFr0xc9vqFj0dXdbba91qpepeI8k8fiI+fsY=rqGqVepae9pg0db9vqaiVgFr0xfr=xfr=xc9adbaqaaeGacaGaaiaabeqaaeqabiWaaaGcbaGafmiCaaNbaGaacqGGOaakcqWG4baEcqGGPaqkcqGH9aqpjuaGdaWcaaqaaiqbdchaWzaaiaGaeiikaGIaemyqae0aaSbaaeaacqqGUbGBaeqaaiabcIcaOiabdIha4jabcMcaPiabcMcaPiabgUcaRiqbdchaWzaaiaGaeiikaGIaemyqae0aaSbaaeaacqqGVbWBaeqaaiabcIcaOiabdIha4jabcMcaPiabcMcaPaqaaiabfo5ahnaaBaaabaGaeeOBa4gabeaacqGHRaWkcqqHtoWrdaWgaaqaaiabb+gaVbqabaaaaaaa@4BD0@

which can be differentiated *k *times to express the *k*th derivative in terms of lower derivatives,

∂xkp(1)[1−Γnk+ΓokΓn+Γo]=∑m=[k/2]kαm∂xmp(1)
 MathType@MTEF@5@5@+=feaafiart1ev1aaatCvAUfKttLearuWrP9MDH5MBPbIqV92AaeXatLxBI9gBaebbnrfifHhDYfgasaacPC6xNi=xI8qiVKYPFjYdHaVhbbf9v8qqaqFr0xc9vqFj0dXdbba91qpepeI8k8fiI+fsY=rqGqVepae9pg0db9vqaiVgFr0xfr=xfr=xc9adbaqaaeGacaGaaiaabeqaaeqabiWaaaGcbaGaeyOaIy7aa0baaSqaaiabdIha4bqaaiabdUgaRbaakiabdchaWjabcIcaOiabigdaXiabcMcaPmaadmaabaGaeGymaeJaeyOeI0scfa4aaSaaaeaacqqHtoWrdaqhaaqaaiabb6gaUbqaaiabdUgaRbaacqGHRaWkcqqHtoWrdaqhaaqaaiabb+gaVbqaaiabdUgaRbaaaeaacqqHtoWrdaWgaaqaaiabb6gaUbqabaGaey4kaSIaeu4KdC0aaSbaaeaacqqGVbWBaeqaaaaaaOGaay5waiaaw2faaiabg2da9maaqahabaacciGae8xSde2aaSbaaSqaaiabd2gaTbqabaGccqGHciITdaqhaaWcbaGaemiEaGhabaGaemyBa0gaaOGaemiCaaNaeiikaGIaeGymaeJaeiykaKcaleaacqWGTbqBcqGH9aqpcqGGBbWwcqWGRbWAcqGGVaWlcqaIYaGmcqGGDbqxaeaacqWGRbWAa0GaeyyeIuoaaaa@61DA@

where the coefficients *α*_*m *_≡ *α*_*m*_(*γ*_n_, *γ*_o_, *γ*) are all positive from the definition (9).

The finite or infinite nature of ∂xkp(1)
 MathType@MTEF@5@5@+=feaafiart1ev1aaatCvAUfKttLearuWrP9MDH5MBPbIqV92AaeXatLxBI9gBaebbnrfifHhDYfgasaacPC6xNi=xH8viVGI8Gi=hEeeu0xXdbba9frFj0xb9qqpG0dXdb9aspeI8k8fiI+fsY=rqGqVepae9pg0db9vqaiVgFr0xfr=xfr=xc9adbaqaaeGacaGaaiaabeqaaeqabiWaaaGcbaGaeyOaIy7aa0baaSqaaiabdIha4bqaaiabdUgaRbaakiabdchaWjabcIcaOiabigdaXiabcMcaPaaa@3453@ depends on the two parameters Γ_n _and Γ_o _and defines the form of the limit degree distribution. The phase diagram Fig. 3A summarizes in the plane (Γ_o _+ Γ_n_, Γ_o _- Γ_n_) the different regimes for the asymptotic degree distribution *p*_*k*_. Γ_o _+ Γ_n _is the global growth rate of the network (Γ_o _+ Γ_n _> 1 to ensure a growing network) and Γ_o _- Γ_n _corresponds to the divergence asymmetry between duplicated proteins. We now discuss the two main stationary regimes for *p*_*k *_and their biological relevance in the case of Γ_n _≤ Γ_o _(the case Γ_n _≤ Γ_o _is deduced by permutating indices):

### • Non-conserved, exponential regime

If both Γ_o _< 1 and Γ_n _< 1, then,

Γnk+Γok<Γn+Γo,for allk≥2
 MathType@MTEF@5@5@+=feaafiart1ev1aaatCvAUfKttLearuWrP9MDH5MBPbIqV92AaeXatLxBI9gBaebbnrfifHhDYfgasaacPC6xNi=xI8qiVKYPFjYdHaVhbbf9v8qqaqFr0xc9vqFj0dXdbba91qpepeI8k8fiI+fsY=rqGqVepae9pg0db9vqaiVgFr0xfr=xfr=xc9adbaqaaeGacaGaaiaabeqaaeqabiWaaaGcbaqbaeqabeWaaaqaaiabfo5ahnaaDaaaleaacqqGUbGBaeaacqWGRbWAaaGccqGHRaWkcqqHtoWrdaqhaaWcbaGaee4Ba8gabaGaem4AaSgaaOGaeyipaWJaeu4KdC0aaSbaaSqaaiabb6gaUbqabaGccqGHRaWkcqqHtoWrdaWgaaWcbaGaee4Ba8gabeaakiabcYcaSaqaaiabbAgaMjabb+gaVjabbkhaYjabbccaGiabbggaHjabbYgaSjabbYgaSbqaaiabdUgaRjabgwMiZkabikdaYaaaaaa@4BA8@

and the factor in front of ∂xkp(1)
 MathType@MTEF@5@5@+=feaafiart1ev1aaatCvAUfKttLearuWrP9MDH5MBPbIqV92AaeXatLxBI9gBaebbnrfifHhDYfgasaacPC6xNi=xH8viVGI8Gi=hEeeu0xXdbba9frFj0xb9qqpG0dXdb9aspeI8k8fiI+fsY=rqGqVepae9pg0db9vqaiVgFr0xfr=xfr=xc9adbaqaaeGacaGaaiaabeqaaeqabiWaaaGcbaGaeyOaIy7aa0baaSqaaiabdIha4bqaaiabdUgaRbaakiabdchaWjabcIcaOiabigdaXiabcMcaPaaa@3453@ in (15) is always strictly positive, which implies that all derivatives of the limit degree distribution are finite. Hence, in this case, the limit degree distribution decreases more rapidly than any power law (see explicit asymptotic development in [47]). Note that this "exponential" regime occurs when the links emerging from each node (Fig. 2) are more likely lost than duplicated at each round of global duplication (as Γ_i _= *γ *+ *γ*_i _< 1 is equivalent to *δδ*_i _> *γγ*_i_). This implies that most nodes eventually disappear, and with them all traces of network evolution, after just a few rounds of global duplication. The network topology is *not *conserved, but instead continuously renewed from duplication of the (few) most connected nodes. From a speciation perspective, this implies that all nodes of a given PPI network realization are eventually more closely related to one another than to any other node of a different PPI network realization, *i.e*. from a different species. Clearly, this class of evolutionary non-conserved PPI networks doest not appear to be biologically relevant, given the typical degree of conservation between orthologous proteins across living kingdoms. As a consequence, we can also conclude from the phase diagram Fig. 3A that exponential PPI networks arising through genome duplication would necessary correspond to non-conserved networks and would thus be presumably irrelevant from a biological perspective. This result actually holds, beyond genome duplication, for evolutionary duplication-divergence dynamics at *any *genomic scale (from single gene to whole genome) and even with *variations *in all evolutionary parameters {γi(n)}
 MathType@MTEF@5@5@+=feaafiart1ev1aaatCvAUfKttLearuWrP9MDH5MBPbIqV92AaeXatLxBI9gBaebbnrfifHhDYfgasaacPC6xNi=xH8viVGI8Gi=hEeeu0xXdbba9frFj0xb9qqpG0dXdb9aspeI8k8fiI+fsY=rqGqVepae9pg0db9vqaiVgFr0xfr=xfr=xc9adbaqaaeGacaGaaiaabeqaaeqabiWaaaGcbaGaei4EaShcciGae83SdC2aa0baaSqaaiabbMgaPbqaaiabcIcaOiabd6gaUjabcMcaPaaakiabc2ha9baa@3528@ at each duplication-divergence process *n*, see [47]. Hence, only non-exponential topologies of PPI networks are likely to be observed in Nature. This corresponds to the second regime discussed below.

### • Conserved, scale-free regime

If Γ_o _> 1 > Γ_n_, then the factor in front of ∂xkp(1)
 MathType@MTEF@5@5@+=feaafiart1ev1aaatCvAUfKttLearuWrP9MDH5MBPbIqV92AaeXatLxBI9gBaebbnrfifHhDYfgasaacPC6xNi=xH8viVGI8Gi=hEeeu0xXdbba9frFj0xb9qqpG0dXdb9aspeI8k8fiI+fsY=rqGqVepae9pg0db9vqaiVgFr0xfr=xfr=xc9adbaqaaeGacaGaaiaabeqaaeqabiWaaaGcbaGaeyOaIy7aa0baaSqaaiabdIha4bqaaiabdUgaRbaakiabdchaWjabcIcaOiabigdaXiabcMcaPaaa@3453@ in (15) can become negative. However, since the generating function should have all its derivatives positive, a negative value for one of them means that it simply does not exist. In fact, for Γ_n _ln Γ_n _+ Γ_o _ln Γ_o _≥ 0 (red line in Fig. 3A and [47]), there is an integer *r *≥ 1 such that,

Γnr+Γor≤Γn+Γo<Γnr+1+Γor+1
 MathType@MTEF@5@5@+=feaafiart1ev1aaatCvAUfKttLearuWrP9MDH5MBPbIqV92AaeXatLxBI9gBaebbnrfifHhDYfgasaacPC6xNi=xI8qiVKYPFjYdHaVhbbf9v8qqaqFr0xc9vqFj0dXdbba91qpepeI8k8fiI+fsY=rqGqVepae9pg0db9vqaiVgFr0xfr=xfr=xc9adbaqaaeGacaGaaiaabeqaaeqabiWaaaGcbaGaeu4KdC0aa0baaSqaaiabb6gaUbqaaiabdkhaYbaakiabgUcaRiabfo5ahnaaDaaaleaacqqGVbWBaeaacqWGYbGCaaGccqGHKjYOcqqHtoWrdaWgaaWcbaGaeeOBa4gabeaakiabgUcaRiabfo5ahnaaBaaaleaacqqGVbWBaeqaaOGaeyipaWJaeu4KdC0aa0baaSqaaiabb6gaUbqaaiabdkhaYjabgUcaRiabigdaXaaakiabgUcaRiabfo5ahnaaDaaaleaacqqGVbWBaeaacqWGYbGCcqGHRaWkcqaIXaqmaaaaaa@4CDE@

implying that all derivatives ∂xkp(1)
 MathType@MTEF@5@5@+=feaafiart1ev1aaatCvAUfKttLearuWrP9MDH5MBPbIqV92AaeXatLxBI9gBaebbnrfifHhDYfgasaacPC6xNi=xH8viVGI8Gi=hEeeu0xXdbba9frFj0xb9qqpG0dXdb9aspeI8k8fiI+fsY=rqGqVepae9pg0db9vqaiVgFr0xfr=xfr=xc9adbaqaaeGacaGaaiaabeqaaeqabiWaaaGcbaGaeyOaIy7aa0baaSqaaiabdIha4bqaaiabdUgaRbaakiabdchaWjabcIcaOiabigdaXiabcMcaPaaa@3453@ are finite up to the *r*th order, while ∂xr+1p(1)
 MathType@MTEF@5@5@+=feaafiart1ev1aaatCvAUfKttLearuWrP9MDH5MBPbIqV92AaeXatLxBI9gBaebbnrfifHhDYfgasaacPC6xNi=xH8viVGI8Gi=hEeeu0xXdbba9frFj0xb9qqpG0dXdb9aspeI8k8fiI+fsY=rqGqVepae9pg0db9vqaiVgFr0xfr=xfr=xc9adbaqaaeGacaGaaiaabeqaaeqabiWaaaGcbaGaeyOaIy7aa0baaSqaaiabdIha4bqaaiabdkhaYjabgUcaRiabigdaXaaakiabdchaWjabcIcaOiabigdaXiabcMcaPaaa@3633@ is infinite. This justifies the following asymptotic expansion of *p*(*x*) in the vicinity of *x *= 1,

*p*(*x*) = 1 - *A*_1_(1 - *x*) - ... - *A*_*r*_(1 - *x*)^*r *^- *A*_*α*_(1 - *x*)^*α *^- ...

for some appropriate *r *<*α *<*r *+ 1. This anzats is then inserted in (14) using (*γx *+ *δ*)(*γ*_n,o_*x *+ *δ*_n,o_) = 1 - Γ_n,o_(1 - *x*) + *γγ*_n,o_(1 - *x*)^2 ^to obtain an equation on the coefficients *A*_1_,...*A*_*r*_. The term *A*_*α *_does not mix with previous terms and gives the following equation for *α*,

Γnα+Γoα=Γn+Γo
 MathType@MTEF@5@5@+=feaafiart1ev1aaatCvAUfKttLearuWrP9MDH5MBPbIqV92AaeXatLxBI9gBaebbnrfifHhDYfgasaacPC6xNi=xI8qiVKYPFjYdHaVhbbf9v8qqaqFr0xc9vqFj0dXdbba91qpepeI8k8fiI+fsY=rqGqVepae9pg0db9vqaiVgFr0xfr=xfr=xc9adbaqaaeGacaGaaiaabeqaaeqabiWaaaGcbaGaeu4KdC0aa0baaSqaaiabb6gaUbqaaGGaciab=f7aHbaakiabgUcaRiabfo5ahnaaDaaaleaacqqGVbWBaeaacqWFXoqyaaGccqGH9aqpcqqHtoWrdaWgaaWcbaGaeeOBa4gabeaakiabgUcaRiabfo5ahnaaBaaaleaacqqGVbWBaeqaaaaa@3E2B@

The limit degree distribution follows a power law in this case,

*p*_*k *_∝ *k*^-*α*-1^

When Γnr+Γor=Γn+Γo
 MathType@MTEF@5@5@+=feaafiart1ev1aaatCvAUfKttLearuWrP9MDH5MBPbIqV92AaeXatLxBI9gBaebbnrfifHhDYfgasaacPC6xNi=xH8viVGI8Gi=hEeeu0xXdbba9frFj0xb9qqpG0dXdb9aspeI8k8fiI+fsY=rqGqVepae9pg0db9vqaiVgFr0xfr=xfr=xc9adbaqaaeGacaGaaiaabeqaaeqabiWaaaGcbaGaeu4KdC0aa0baaSqaaiabb6gaUbqaaiabdkhaYbaakiabgUcaRiabfo5ahnaaDaaaleaacqqGVbWBaeaacqWGYbGCaaGccqGH9aqpcqqHtoWrdaWgaaWcbaGaeeOBa4gabeaakiabgUcaRiabfo5ahnaaBaaaleaacqqGVbWBaeqaaaaa@3D77@ for exactly some integer *r *≥ 1 the last term in Eq. 18 should be replaced by (1 - *x*)^*r *^ln(1 - *x*), and the limit degree distribution decreases like *k*^-*r*-1 ^(see red and blue "exponent" lines in Fig. 3A for *α *+ 1 = 2, 3, 4, ...)

Note that scale-free degree distributions emerge under successive, global network duplications only if the "old" node copy has its links more likely duplicated than lost at each round of global duplication (as Γ_o _= *γ *+ *γ*_o _> 1 is equivalent to *γγ*_o _> *δδ*_o_). Thus, "old" nodes statistically keep on increasing their connectivity once they have emerged as "new" nodes by duplication. From biological perspective, this implies that most nodes and their surrounding links are conserved *throughout *the evolution process, thereby ensuring that local topologies of previous networks remain embedded in subsequent networks.

In summary, whole genome duplication with asymmetric divergence of duplicated proteins leads to the emergence of two classes of PPI networks with finite asymptotic degree distributions : *i) *PPI networks with an exponential degree distribution and without protein nor topology evolutionary conservation and *ii) *PPI networks with a scale-free limit degree distribution and protein conservation together with at least some local topology conservation. All other evolution scenarios, which do not lead to finite asymptotic degree distributions, are unlikely to model biologically relevant cases; they correspond either to an *exponential *disappearance of the whole PPI network (*i.e*. if Γ_n _+ Γ_o _< 1) or to an *exponential *shift of *all *proteins towards higher and higher connectivities (*i.e*. dense regime in Fig. 3A for Γ_n_Γ_o _> 1) [47]. Hence, from a biological perspective, evolutionary conservation and scale-free topology of PPI networks are intrinsically linked under genome duplication. Evolutionary conservation, which is a fundamental property of proteins and PPI networks (see *e.g*. Fig. 1) is shown to necessary lead to scale-free PPI network topologies. It is, in fact, a very general and fundamental result that is not sensitive to *variations *in the model parameters {γi(n)}
 MathType@MTEF@5@5@+=feaafiart1ev1aaatCvAUfKttLearuWrP9MDH5MBPbIqV92AaeXatLxBI9gBaebbnrfifHhDYfgasaacPC6xNi=xH8viVGI8Gi=hEeeu0xXdbba9frFj0xb9qqpG0dXdb9aspeI8k8fiI+fsY=rqGqVepae9pg0db9vqaiVgFr0xfr=xfr=xc9adbaqaaeGacaGaaiaabeqaaeqabiWaaaGcbaGaei4EaShcciGae83SdC2aa0baaSqaaiabbMgaPbqaaiabcIcaOiabd6gaUjabcMcaPaaakiabc2ha9baa@3528@ on the evolutionary time scale *n *and also holds for duplication-divergence events at *any *genomic scale from single gene to whole genome duplication (see [47]). In other words, scale-free topologies of PPI networks appear to be a simple consequence of the evolutionary conservation of PPI networks and their underlying proteins.

## Abbreviations

*WGD *: *W*hole *G*enome *D*uplication; *PPI *network : *P*rotein-*P*rotein *I*nteraction network.

## Authors' contributions

HI conceived the research, KE and HI performed the research and wrote the paper.

## Supplementary Material

Additional File 1 Supporting Information (6 pages). I. Model of PPI network evolution under WGD with symmetric divergence and link "complementation". II. Proof of Functional Recurrences (Eq. 8 and Eq. S1). III. Gene functionalization patterns in different models of PPI network evolution under WGD. IV. Statistical weighting of indirect interactions from protein complexes. V. Evolution of PPI networks including self-interacting proteins under WGD. Click here for file

## References

[B1] Li WH (1997). Molecular Evolution.

[B2] Ohno S (1970). Evolution by Gene Duplication.

[B3] Sparrow AH, Naumann AF (1976). Evolution of genome size by DNA doublings. Science.

[B4] Wolfe KH, Shields DC (1997). Molecular evidence for an ancient duplication of the entire yeast genome. Nature.

[B5] McLysaght A, Hokamp K, Wolfe KH (2002). Extensive genomic duplication during early chordate evolution. Nat Genet.

[B6] Wong S, Butler G, Wolfe KH (2002). Gene order evolution and paleopolyploidy in hemiascomycete yeasts. Proc Natl Acad Sci USA.

[B7] Simillion C, Vandepoele K, Van Montagu MCE, Zabeau M, Van de Peer Y (2002). The hidden duplication past of Arabidopsis thaliana. Proc Natl Acad Sci USA.

[B8] Blanc G, Hokamp K, Wolfe KH (2003). A recent polyploidy superimposed on older large-scale duplications in the Arabidopsis genome. Genome Res.

[B9] Kellis M, Birren BW, Lander ES (2004). Proof and evolutionary analysis of ancient genome duplication in the yeast Saccharomyces cerevisiae. Nature.

[B10] Dujon B, Sherman D, Fischer G, Durrens P, Casaregola S, Lafontaine I, De Montigny J, Marck C, Neuveglise C, Talla E, Goffard N, Frangeul L, Aigle M, Anthouard V, Babour A, Barbe V, Barnay S, Blanchin S, Beckerich JM, Beyne E, Bleykasten C, Boisrame A, Boyer J, Cattolico L, Confanioleri F, De Daruvar A, Despons L, Fabre E, Fairhead C, Ferry-Dumazet H, Groppi A, Hantraye F, Hennequin C, Jauniaux N, Joyet P, Kachouri R, Kerrest A, Koszul R, Lemaire M, Lesur I, Ma L, Muller H, Nicaud JM, Nikolski M, Oztas S, Ozier-Kalogeropoulos O, Pellenz S, Potier S, Richard GF, Straub ML, Suleau A, Swennen D, Tekaia F, Wesolowski-Louvel M, Westhof E, Wirth B, Zeniou-Meyer M, Zivanovic I, Bolotin-Fukuhara M, Thierry A, Bouchier C, Caudron B, Scarpelli C, Gaillardin C, Weissenbach J, Wincker P, Souciet JL (2004). Genome evolution in yeasts. Nature.

[B11] Jaillon O, Aury JM, Brunet F, Petit JL, Stange-Thomann N, Mauceli E, Bouneau L, Fischer C, Ozouf-Costaz C, Bernot A, Nicaud S, Jaffe D, Fisher S, Lutfalla G, Dossat C, Segurens B, Dasilva C, Salanoubat M, Levy M, Boudet N, Castellano S, Anthouard V, Jubin C, Castelli V, Katinka M, Vacherie B, Biemont C, Skalli Z, Cattolico L, Poulain J, De Berardinis V, Cruaud C, Duprat S, Brottier P, Coutanceau JP, Gouzy J, Parra G, Lardier G, Chapple C, McKernan KJ, McEwan P, Bosak S, Kellis M, Volff JN, Guigo R, Zody MC, Mesirov J, Lindblad-Toh K, Birren B, Nusbaum C, Kahn D, Robinson-Rechavi M, Laudet V, Schachter V, Quetier F, Saurin W, Scarpelli C, Wincker P, Lander ES, Weissenbach J, Roest Crollius H (2004). Genome duplication in the teleost fish Tetraodon nigroviridis reveals the early vertebrate proto-karyotype. Nature.

[B12] Dehal P, Boore JL (2005). Two rounds of whole genome duplication in the ancestral vertebrate. PLoS Biol.

[B13] Scannell DR, Byrne KP, Gordon JL, Wong S, Wolfe KH (2006). Multiple rounds of speciation associated with reciprocal gene loss in polyploid yeasts. Nature.

[B14] Doolittle RF (1995). The multiplicity of domains in proteins. Annu Rev Biochem.

[B15] Riley M, Labedan B (1997). Protein evolution viewed throughE.coli protein sequences: introducing the notion of a structural segment of homology, the module. J Mol Biol.

[B16] Tatusov RL, Koonin EV, Lipman DJ (1997). A genomic perspectiveon protein families. Science.

[B17] Koonin EV, Aravind L, Kondrashov AS (2000). The impact of comparative genomics on our understanding of evolution. Cell.

[B18] Apic G, Gough J, Teichmann SA (2001). Domain combinations in archaeal, eubacterial and eukaryotic proteomes. J Mol Biol.

[B19] Orengo CA, Thornton JM (2005). Protein families and their evolution-a structural perspective. Annual Rev Biochem.

[B20] Bornberg-Bauer E, Beaussart F, Kummerfeld SK, Teichmann SA, Weiner J (2005). The evolution of domain arrangements in proteins and interaction networks. Cell Mol Life Sci.

[B21] Lynch M, Force A (2000). Gene duplication and the origin of interspecific genomic incompatibility. Am Nat.

[B22] Adams KL, Wendel JF (2005). Polyploidy and genome evolution in plants. Current Opinion in Plant Biology.

[B23] Tuskan GA, DiFazio S, Jansson S, Bohlmann J, Grigoriev I, Hellsten U, Putnam N, Ralph S, Rombauts S, Salamov A, Schein J, Sterck L, Aerts A, Bhalerao RR, Bhalerao RP, Blaudez D, Boerjan W, Brun A, Brunner A, Busov V, Campbell M, Carlson J, Chalot M, Chapman J, Chen GL, Cooper D, Coutinho PM, Couturier J, Covert S, Cronk Q, Cunningham R, Davis J, Degroeve S, Déjardin A, dePamphilis C, Detter J, Dirks B, Dubchak I, Duplessis S, Ehlting J, Ellis B, Gendler K, Goodstein D, Gribskov M, Grimwood J, Groover A, Gunter L, Hamberger B, Heinze B, Helariutta Y, Henrissat B, Holligan D, Holt R, Huang W, Islam-Faridi N, Jones S, Jones-Rhoades M, Jorgensen R, Joshi C, Kangasjärvi J, Karlsson J, Kelleher C, Kirkpatrick R, Kirst M, Kohler A, Kalluri U, Larimer F, Leebens-Mack J, Leplé JC, Locascio P, Lou Y, Lucas S, Martin F, Montanini B, Napoli C, Nelson DR, Nelson C, Nieminen K, Nilsson O, Pereda V, Peter G, Philippe R, Pilate G, Poliakov A, Razumovskaya J, Richardson P, Rinaldi C, Ritland K, Rouzé P, Ryaboy D, Schmutz J, Schrader J, Segerman B, Shin H, Siddiqui A, Sterky F, Terry A, Tsai CJ, Uberbacher E, Unneberg P, Vahala J, Wall K, Wessler S, Yang G, Yin T, Douglas C, Marra M, Sandberg G, de Peer YV, Rokhsar D (2006). The Genome of Black Cottonwood, Populus trichocarpa (Torr. & Gray). Science.

[B24] Panopoulou G, Hennig S, Groth D, Krause A, Poustka AJ, Herwig R, Vingron M, Lehrach H (2003). New evidence for genome-wide duplications at the origin of vertebrates using an amphioxus gene set and completed animal genomes. Genome Res.

[B25] David L, Blum S, Feldman MW, Lavi U, Hillel J (2003). Recent duplication of the common carp (Cyprinus carpio L.) genome as revealed by analyses of microsatellite loci. Mol Biol Evol.

[B26] Gallardo MH, Gonzalez CA, Cebrian I (2006). Molecular cytogenetics and allotetraploidy in the red vizcacha rat, Tympanoctomys barrerae (Rodentia, Octodontidae). Genomics.

[B27] Guc-Scekic M, Milasin J, Stevanovic M, Stojanov LJ, Djordjevic M (2002). Tetraploidy in a 26-month-old girl (cytogenetic and molecular studies). Clin Genet.

[B28] Gambi MC, Ramella L, Sella G, Protto P, Aldieri E (1997). Variation in genome size in benthic polychaetes: systematic and ecological relationships. Journal Marine Biological Association UK.

[B29] Gregory TR, Hebert PD, Kolasa J (2000). Evolutionary implications of the relationship between genome size and body size in flatworms and copepods. Heredity.

[B30] Guo XM, Allen SK (1995). The successful induction of tetraploidy in the Pacific oyster *Crassostrea gigas *(Thunberg). Aquaculture.

[B31] Grozeva S, Kuznetsova VG, Nokkala S (2004). Patterns of chromosome banding in four nabid species (Heteroptera, Cimicomorpha, Nabidae) with high chromosome number karyotypes. Hereditas.

[B32] Dufresne F, Hebert PDN (1995). Polyploidy and clonal diversity in an arctic cladoceran. Heredity.

[B33] Aury J, Jaillon O, Duret L, Noel B, Jubin C, Porcel B, Segurens B, Daubin V, Anthouard V, Aiach N, Arnaiz O, Billaut A, Beisson J, Blanc I, Bouhouche K, Camara F, Duharcourt S, Guigo R, Gogendeau D, Katinka M, Keller A, Kissmehl R, Klotz C, Koll F, Le Mouel A, Lepere G, Malinsky S, Nowacki M, Nowak J, Plattner H, Poulain J, Ruiz F, Serrano V, Zagulski M, Dessen P, Betermier M, Weissenbach J, Scarpelli C, Schachter V, Sperling L, Meyer E, Cohen J, Wincker P (2006). Global trends of whole-genome duplications revealed by the ciliate Paramecium tetraurelia. Nature.

[B34] Grandjean V, Hauck Y, Le Derout J, Hirschbein L (1996). Noncomplementing Diploids From Bacillus subtilis Protoplast Fusion: Relationship Between Maintenance of Chromosomal Inactivation and Segregation Capacity. Genetics.

[B35] Itaya M, Tsuge K, Koizumi M, Fujita K, Kagaku M (2005). Combining two genomes in one cell: Stable cloning of the Synechocystis PCC6803 genome in the Bacillus subtilis 168 genome. Proc Natl Acad Sci USA.

[B36] Vázquez A, Flammini A, Maritan A, Vespignani A (2003). Modeling of protein interaction networks. ComPlexUs.

[B37] Middendorf M, Ziv E, Wiggins C (2005). Inferring network mechanisms: the Drosophila melanogaster protein interaction network. Proc Natl Acad Sci USA.

[B38] Alfarano C, Andrade CE, Anthony K, Bahroos N, Bajec M, Bantoft K, Betel D, Bobechko B, Boutilier K, Burgess E, Buzadzija K, Cavero R, D'Abreo C, Donaldson I, Dorairajoo D, Dumontier MJ, Dumontier MR, Earles V, Farrall R, Feldman H, Garderman E, Gong Y, Gonzaga R, Grytsan V, Gryz E, Gu V, Haldorsen E, Halupa A, Haw R, Hrvojic A, Hurrell L, Isserlin R, Jack F, Juma F, Khan A, Kon T, Konopinsky S, Le V, Lee E, Ling S, Magidin M, Moniakis J, Montojo J, Moore S, Muskat B, Ng I, Paraiso JP, Parker B, Pintilie G, Pirone R, Salama JJ, Sgro S, Shan T, Shu Y, Siew J, Skinner D, Snyder K, Stasiuk R, Strumpf D, Tuekam B, Tao S, Wang Z, White M, Willis R, Wolting C, Wong S, Wrong A, Xin C, Yao R, Yates B, Zhang S, Zheng K, Pawson T, Ouellette BFF, Hogue CWV (2005). The Biomolecular Interaction Network Database and related tools 2005 update. Nucleic Acids Res.

[B39] Mewes HW, Frishman D, Mayer K, Münsterkötter M, Noubibou O, Pagel P, Rattei T, Oesterheld M, Ruepp A, Stumpflen V (2005). MIPS: analysis and annotation of proteins from whole genomes in 2005. Nucleic Acids Res.

[B40] Maslov S, Sneppen K, Eriksen KA, Yan KK (2004). Upstream plasticity and downstream robustness in evolution of molecular networks. BMC Evol Biol.

[B41] Albert R, Barabási AL (2002). Statistical Mechanics of Complex Networks. Rev Mod Phys.

[B42] Barabási AL, Oltvai ZN (2004). Network Biology. Nat Rev Genetics.

[B43] Raval A (2003). Some asymptotic properties of duplication graphs. Phys Rev E Stat Nonlin Soft Matter Phys.

[B44] Berg J, Lässig M, Wagner A (2004). Structure and evolution of protein interaction networks: a statistical model for link dynamics and gene duplications. BMC Evol Biol.

[B45] Ispolatov I, Krapivsky PL, Yuryev A (2005). Duplication-divergence model of protein interaction network. Phys Rev E Stat Nonlin Soft Matter Phys.

[B46] Hartl DL (2000). Molecular melodies in high and low C. Nat Rev Genet.

[B47] Evlampiev K, Isambert H (2006). Asymptotic Evolution of Protein-Protein Interaction Networks for General Duplication-Divergence Models. preprint.

[B48] Conant GC, Wolfe KH (2006). Functional partitioning of yeast co-expression networks after genome duplication. PLoS Biol.

[B49] Flajolet P, Sedgewick R (2006). Analytic Combinatorics.

[B50] Maslov S, Sneppen K (2002). Specificity and stability in topology of protein networks. Science.

[B51] Kondrashov FA, Rogozin IB, Wolf YI, Koonin EV (2002). Selection in the evolution of gene duplications. Genome Biol.

[B52] Zhang P, Gu Z, Li WH (2003). Different evolutionary patterns between young duplicate genes in the human genome. Genome Biol.

[B53] Conant GC, Wagner A (2003). Asymmetric sequence divergence of duplicate genes. Genome Res.

[B54] Fares MA, Byrne KP, Wolfe KH (2006). Rate asymmetry after genome duplication causes substantial long-branch attraction artifacts in the phylogeny of Saccharomyces species. Mol Biol Evol.

[B55] Doolittle RF (2005). Evolutionary aspects of whole-genome biology. Curr Opin Struct Biol.

[B56] Uetz P, Giot L, Cagney G, Mansfield T, Judson R, Knight J, Lockshon D, Narayan V, Srinivasan M, Pochart P, Qureshi-Emili A, Li Y, Godwin B, Conover D, Kalbfleisch T, Vijayadamodar G, Yang M, Johnston M, Fields S, Rothberg J (2000). A comprehensive analysis of protein-protein interactions in Saccharomyces cerevisiae. Nature.

[B57] Dokholyan NV, Shakhnovich B, Shakhnovich EI (2002). Expanding protein universe and its origin from the biological Big Bang. Proc Natl Acad Sci USA.

[B58] Spirin V, Mirny LA (2003). Protein complexes and functional modules in molecular networks. Proc Natl Acad Sci USA.

[B59] Wuchty S, Oltvai ZN, Barabási AL (2003). Evolutionary conservation of motif constituents in the yeast protein interaction network. Nat Genet.

[B60] Wuchty S (2004). Evolution and topology in the yeast protein interaction network. Genome Res.

[B61] Vergassola M, Vespignani A, Dujon B (2005). Cooperative evolution in protein complexes of yeast from comparative analyses of its interaction network. Proteomics.

[B62] Hartwell LH, Hopfield JJ, Leibler S, Murray AW (1999). From molecular to modular cell biology. Nature.

[B63] Milo R, Shen-Orr S, Itzkovitz S, Kashtan N, Chklovskii D, Alon U (2002). Network motifs: simple building blocks of complex networks. Science.

[B64] Guelzim N, Bottani S, Bourgine P, Képès F (2002). Topological and causal structure of the yeast transcriptional regulatory Genet.

[B65] Yeger-Lotem E, Sattath S, Kashtan N, Itzkovitz S, Milo R, Pinter RY, Alon U, Margalit H (2004). Network motifs in integrated cellular networks of transcription-regulation and protein-protein interaction. Proc Natl Acad Sci USA.

[B66] Francois P, Hakim V (2004). Design of genetic networks with specified functions by evolution in silico. Proc Natl Acad Sci USA.

[B67] Berg J, Lässig M (2004). Local graph alignment and motif search in biological networks. Proc Natl Acad Sci USA.

[B68] Prill RJ, Iglesias PA, Levchenko A (2005). Dynamic properties of network motifs contribute to biological network organization. PLoS Biol.

[B69] Mazurie A, Bottani S, Vergassola M (2005). An evolutionary and functional assessment of regulatory network motifs. Genome Biol.

[B70] Buchler NE, Gerland U, Hwa T (2005). Nonlinear protein degradation and the function of genetic circuits. Proc Natl Acad Sci USA.

[B71] Gelfand MS (2006). Evolution of transcriptional regulatory networks in microbial genomes. Curr Opin Struct Biol.

[B72] Birchler JA, Bhadra U, Bhadra MP, Auger DL (2001). Dosage-dependent gene regulation in multicellular eukaryotes: implications for dosage compensation, aneuploid syndromes, and quantitative traits. Dev Biol.

[B73] Murzin AG, Brenner SE, Hubbard T, Chothia C (1995). SCOP: a structural classification of proteins database for the investigation of sequences and structures. J Mol Biol.

[B74] Gough J, Karplus K, Hughey R, Chothia C (2001). Assignment of Homology to Genome Sequences using a Library of Hidden Markov Models that Represent all Proteins of Known Structure. J Mol Biol.

[B75] Gavin AC, Bosche M, Krause R, Grandi P, Marzioch M, Bauer A, Schultz J, Rick JM, Michon AM, Cruciat CM (2002). Functional organization of the yeast proteome by systematic analysis of protein complexes. Nature.

[B76] Ho Y, Gruhler A, Heilbut A, Bader GD, Moore L, Adams SL, Millar A, Taylor P, Bennett K, Boutilier K (2002). Systematic identification of protein complexes in Saccharomyces cerevisiae by mass spectrometry. Nature.

[B77] Gavin AC, Aloy P, Grandi P, Krause R, Boesche M, Marzioch M, Rau C, Jensen LJ, Bastuck S, Dumpelfeld B (2006). Proteome survey reveals modularity of the yeast cell machinery. Nature.

[B78] Han JD, Bertin N, Hao T, Goldberg DS, Berriz GF, Zhang LV, Dupuy D, Walhout AJ, Cusick ME, Roth FP, Vidal M (2004). Evidence for dynamically organized modularity in the yeast protein-protein interaction network. Nature.

[B79] Kim PM, Lu LJ, Xia Y, Gerstein MB (2006). Relating three-dimensional structures to protein networks provides evolutionary insights. Science.

[B80] Wolf YI, Brenner SE, Bash PA, Koonin EV (1999). Distribution of protein folds in the three superkingdoms of life. Genome Res.

[B81] Ekman D, Bjorklund AK, Frey-Skott J, Elofsson A (2005). Multi-domain proteins in the three kingdoms of life: orphan domains and other unassigned regions. J Mol Biol.

[B82] Ispolatov I, Yuryev A, Mazo I, Maslov S (2005). Binding properties and evolution of homodimers in protein-protein interaction networks. Nucleic Acids Res.

[B83] Kim WK, Henschel A, Winter C, Schroeder M (2006). The Many Faces of Protein-Protein Interactions: A Compendium of Interface Geometry. PLoS Comput Biol.

[B84] Monod J (1970). Le hazard et la nécessité Seuil.

[B85] Fraser HB, Wall DP, Hirsh AE (2003). A simple dependence between protein evolution rate and the number of protein-protein interactions. BMC Evol Biol.

[B86] Papp B, Pál C, Hurst LD (2003). Dosage sensitivity and the evolution of gene families in yeast. Nature.

[B87] Maere S, De Bodt S, Raes J, Casneuf T, Van Montagu M, Kuiper M, Van de Peer Y (2005). Modeling gene and genome duplications in eukaryotes. Proc Natl Acad Sci USA.

