# Supplementary material for: Modeling protein network evolution under genome duplication and domain shuffling
Source: BMC Syst Biol. 2007 Nov 13;1:49. doi: 10.1186/1752-0509-1-49 (PMC2245809; doi:10.1186/1752-0509-1-49)
Supplement: Additional File 1 — Supporting Information (6 pages). I. Model of PPI network evolution under WGD with symmetric divergence and link "complementation". II. Proof of Functional Recurrences (Eq. 8 and Eq. S1). III. Gene functionalization patterns in different models of PPI network evolution under WGD. IV. Statistical weighting of indirect interactions from protein complexes. V. Evolution of PPI networks including self-interacting proteins under WGD. [file 1752-0509-1-49-S1.pdf]

# Modeling protein network evolution under genome duplication and domain shuffling

Kirill Evlampiev and Hervé Isambert*

CNRS UMR168, Institut Curie, Section de Recherche, 11 rue P. & M. Curie, 75005 Paris, France

*Corresponding author

Email: Kirill Evlampiev - kirill.evlampiev@curie.fr; Hervé Isambert*- herve.isambert@curie.fr;

## SUPPORTING INFORMATION

### I. Model of PPI network evolution under WGD with symmetric divergence and link "complementation"

An alternative model of interest is the so-called "duplication-mutation-complementation" model initially proposed in the context of protein network evolution through successive *local* duplications [1, 2]. This model can be easily adapted to the context of PPI network evolution through whole genome duplication, Fig. S1. After each global duplication step, the probability to keep an instance of each interaction is now distributed randomly over the four equivalent links without reference to particular protein duplicates, unlike in the main text model, Fig. 2. The complementation step (which ensures that at least one instance of each previous link is retained) can be enforced here through the "old" link copy ($\gamma_o = 1$) with $\gamma_n$ corresponding to the "new" interaction sharing no node with $\gamma_o$, while $\gamma$ still pertains to the last two equivalent cross links. This model is thus effectively symmetric from the protein point of view and readily yields the following recurrence for the generating function of the network degree distribution.

$$F^{(n+1)}(x) = 2F^{(n)}\big((\gamma x+\delta)(\gamma_e x+\delta_e)\big), \quad (1)$$

where $\gamma_e = (\gamma_n + \gamma_o)/2$ and $\delta_e = (\delta_n + \delta_o)/2$ are effective average probabilities to retain or delete old and new links (see below for proof details). Hence, the model of PPI network evolution with link complementation is in fact equivalent to the case of a symmetric divergence of duplicated proteins in the previous general model. Such symmetric divergence of duplicated proteins yields either a stationary, non-conserved exponential regime ($\Gamma_n + \Gamma_o < 2$, Fig. 3A) or a non-stationary dense regime [3] ($\Gamma_n + \Gamma_o > 2$, Fig. 3A).

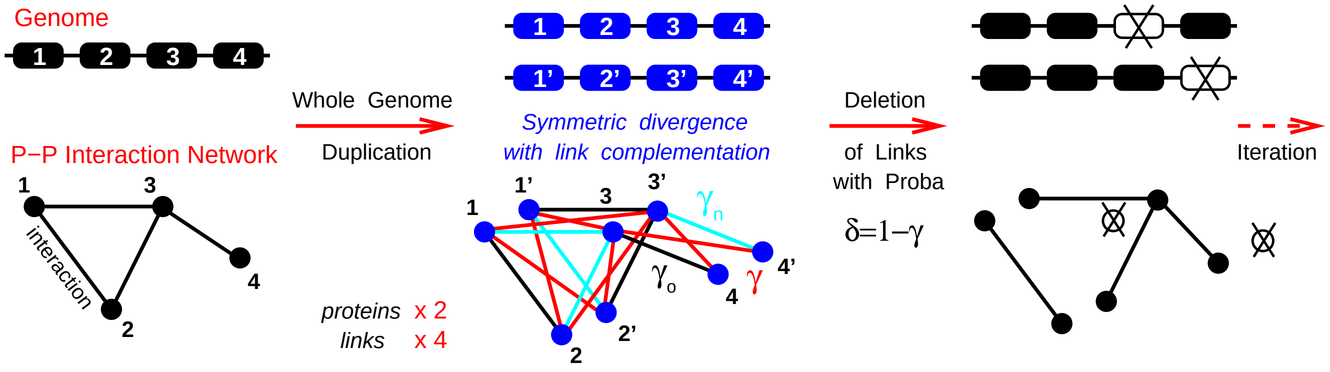


Figure S1. Alternative Model of PPI network evolution through whole genome duplication with *symmetric divergence* of duplicated proteins and *random link "complementation"* [1, 2].

Hence, the "duplication-mutation-complementation" model *cannot* lead to evolutionary conserved PPI networks with scale-free topology, in the context of whole genome duplication evolution, by contrast to the same model applied to local duplication with time-linear evolution [1, 2].

## II. Proof of Functional Recurrences (Eq. 8 and Eq. S1)

After each whole genome duplication, each node has at most doubled its number of neighbors counted through powers of $x$ in the generating function. Hence, a given PPI network realization with $N_k$ nodes of connectivity $k$ ($k \geq 0$) will contribute to the next duplicated ensemble of PPI networks as,

$$N_k x^k \to N_k x^{2k} \tag{2}$$

After link deletion with probability $\delta$ or $\delta_i = \delta_o, \delta_n$, it contributes to the $x^m$ terms of the generating function (with $m = 0, \ldots, 2k$) as,

$$N_k x^{2k} \to N_k \left( \sum_{\ell=0}^{k} \binom{k}{\ell} (\gamma x)^{\ell} \delta^{k-\ell} \right) \left( \sum_{\ell=0}^{k} \binom{k}{\ell} (\gamma_i x)^{\ell} \delta_i^{k-\ell} \right) = N_k \Big( (\gamma x + \delta)(\gamma_i x + \delta_i) \Big)^k \tag{3}$$

for the *asymmetric divergence* model (Fig. 2, Eq. 8, main text) and as,

$$\begin{aligned} N_k x^{2k} \;&\to\; N_k \left( \sum_{\ell=0}^{k} \binom{k}{\ell} (\gamma x)^{\ell} \delta^{k-\ell} \right) \left[ \sum_{j=0}^{k} \binom{k}{j} \left( \sum_{\ell_o=0}^{j} \frac{1}{2^j} \binom{j}{\ell_o} (\gamma_o x)^{\ell_o} \delta_o^{j-\ell_o} \right) \left( \sum_{\ell_n=0}^{k-j} \frac{1}{2^{k-j}} \binom{k-j}{\ell_n} (\gamma_n x)^{\ell_n} \delta_n^{k-j-\ell_n} \right) \right] \\ &\to\; N_k \Big( (\gamma x + \delta)(\gamma_e x + \delta_e) \Big)^k \end{aligned} \tag{4}$$

with $\gamma_e = (\gamma_o + \gamma_n)/2$ and $\delta_e = (\delta_o + \delta_n)/2$ for the *symmetric divergence* model with *link "complementation"* [1,2] (Fig. S1, Eq. S1).

## III. Gene functionalization patterns in different models of PPI network evolution under WGD

The initial model depicted on Fig. 2 with *asymmetric divergence* of duplicated proteins leads typically to "neofunctionalization" of "new" duplicates, while "old" duplicates retain most initial interactions (if not all for $\gamma_o = 1$).

By contrast, the alternative model depicted on Fig. S1 with *symmetric divergence* of duplicated proteins and *random link "complementation"* [1,2] leads typically to random "subfunctionalization" between protein duplicates *at the level of individual interactions*. However, this eventually leads to exponential degree distributions with *no* topology conservation of the PPI network (see above), whereas scale-free degree distributions with at least local topology conservation of the PPI network indeed emerge under the initial asymmetric model, Fig. 2.

Yet, as discussed in the main text, the necessary *asymmetric divergence* of protein duplicates occurs "spontaneously" at the level of protein-binding sites rather than of the entire (multi-domain) proteins, as assumed in Fig. 2. This motivates the redefinition of the initial model in terms of protein-binding domains (Fig. 4A) to capture the *asymmetric divergence* of protein duplicates *at the level of protein-binding sites* and allow, at the same time, for extensive domain shuffling events of multidomain proteins (see main text).

This more elaborate model of PPI network evolution by whole genome duplication and domain shuffling encompasses both "neofunctionalization" and "subfunctionalization" of gene duplicates *at the level of protein domains*, in agreement with the suggestion that gene/protein evolution should be analyzed in terms of domains rather than entire proteins [4–10]. In addition, this combined model of PPI network evolution also provides a theoretical framework to describe the evolution of the "combinatorial logic" behind indirect interactions within multi-protein complexes (see Fig. 4A and main text).

## IV. Statistical weighting of indirect interactions from protein complexes

We use a statistical implemention of the "combinatorial logic" underlying *indirect* protein interactions. Indirect interactions between protein pairs are weighted by the product of binding site "availabilities" along the shortest

weighted path of intermediate direct interactions connecting them. The "availability" $a_i$ of a binding site $i$ is defined as the relative expression level ($e_i$) with respect to its first neighbor binding partners $j$ of connectivity $d_j$ ,

$$a_i = \frac{e_i}{e_i + \sum_{j \in \langle i \rangle} e_j / d_j} < 1 \tag{5}$$

Where expression level $e_j$ can be distributed with specific statistics, such as randomly, uniformly or according to characteristic power laws, as reported experimentally [11–15]. Yet, in practice, we found that the predicted large scale topological features of PPI networks depend only weakly on the specific distribution of expression levels (for reasonable distribution range).

The *statistical probability* of an (intermediate) direct interaction between domains $i$ and $j$ is then proportional to $a_i a_j$, which we use in a Dijkstra-like algorithm [16] for additive distance minimization assigning $d^\circ_{ij} = -\ln(a_i a_j) > 0$ weights between interacting domains $i$ and $j$. Because of the presence of both covalent peptide bonds and direct, noncovalent interactions between protein domains (Fig. 4A), indirect protein-protein interactions correspond to *alternating paths* of noncovalent and covalent interactions *with no successive noncovalent interactions* which are forbidden by the shared binding site constraint (*i.e.* a binding site can only interact with one binding partner at a time). We describe below an algorithm which performs a simultaneous minimization for paths starting with a covalent bond ($c_{ij}$) and paths starting with a direct, noncovalent interaction ($d_{ij}$). (An additional variable for second node $v_{ij}$ on the path is also needed to avoid non-physical "covalent loops".)

The initialization of distances between protein domains is:

$$\begin{array}{ll} c^\circ_{ij} = \text{Max}, \;\; v^\circ_{ij} = j & \text{for all } (i,j) \text{ pairs, and} \\ \delta_{ij} = d^\circ_{ij} = -\ln(a_i a_j) & \text{for direct, noncovalent interactions,} \\ \delta_{ij} = 0, \;\; d^\circ_{ij} = \text{Max} & \text{for covalent bonds,} \\ \delta_{ij} = d^\circ_{ij} = \text{Max} & \text{otherwise.} \end{array}$$

We then iterate until convergence (after $N^2\times$ (longest path) operations):

$$\begin{aligned} d'_{ij} &= \min\big(d_{ij}, \min_{k \in \langle i \rangle_d} (\delta_{ik} + c_{kj})\big) \\ c'_{ij} &= \min\big(c_{ij}, \min_{k \in \langle i \rangle_c, v_{kj} \neq i} (\delta_{ik} + \min(d_{kj}, c_{kj}))\big) \\ v'_{ij} &= \{k \in \langle i \rangle_c \mid v_{kj} \neq i, \; \min(\delta_{ik} + \min(d_{kj}, c_{kj}))\} \end{aligned}$$

and remove eventually the minimum paths starting with a covalent bond (to avoid double counting of indirect interactions for multidomain proteins below):

$$d_{ij} = \text{Max} \qquad \text{if} \;\; d_{ij} \geq \min(c_{ij}, c_{ji}) \tag{6}$$

Hence, the probabilities to observe a *single indirect* interactions within protein complexes is given by:

$$\begin{array}{ll} w_{ij} = 0 & \text{if} \;\; d_{ij} = \text{Max} \\ w_{ij} = \beta \exp(-d_{ij}) & \text{otherwise,} \end{array}$$

with the normalization condition $\Sigma_{i<j} w_{ij} = 1$, which gives $1/\beta = \Sigma_{i<j} \exp(-d_{ij})$.
$w_{ij}$ is thus the normalized product of availabilities $a_k$ along the shortest weighted path between $i$ and $j$.

Finally, the individual probabilities $p_{ij}$ to observe a total of $M$ *indirect* interactions within protein complexes are given by:

$$p_{ij} = 1 - (1 - w_{ij})^n \tag{7}$$

where $n$ is solution of $\Sigma_{i<j} p_{ij} = M$.

Given the number $M$ of indirect interactions in various data sets [17–19], we have assessed their expected contribution to the large scale topology of Yeast PPI network from the two-parameter $\gamma - \lambda$ model described in the main

text. $M \simeq 28,000$ corresponds to the sum of about $9,000$ direct physical interactions from the BIND database [20] (Fig. 3B&C filled symbols) and about $19,000$ "matrix" interactions from [17,18] between $2,100$ proteins already involved in direct physical interactions (out of $4,576$ proteins in the BIND database, Fig. 4C filled symbols). "Matrix" interactions from [19] (Fig. 4C open symbols) are "reconstructed" from supplementary information files of [19] as follows: "matrix" interactions are included for (each complex core)×(each associated "module") and (each complex core)×(each associated "attachment" = one protein). This reconstructed dataset should therefore be considered as incomplete, since "matrix" interactions between compatible modules and/or attachments associated to a given core are *not* taken into account (information not given in [19]).

Numerical fits ($\gamma = 0.1$, $\lambda = 0.3$) are displayed on Fig. 4C (for direct *and* indirect interactions) for both connectivity distribution (green) and average connectivity of first neighbors (blue). They corresponds to the *same* adjusted values ($\gamma = 0.1$, $\lambda = 0.3$) as in Fig. 4B (for direct interactions only).

## V. Evolution of PPI networks including self-interacting proteins under WGD

The possibility of protein homo-oligomerization can be explicitly taken into account by introducing 2 types of nodes corresponding respectively to *i)* self-interacting proteins with self-link loops and *ii)* non-self-interacting proteins without self-link loops, Fig. S2. Available data on PPI networks reveals that about 10 to 15% of interacting proteins are self-interacting [20]. In principle, the detailed evolution of PPI network conservation and topology is affected by self-link loops which provide a source of duplication-derived *de novo* interactions between "old" and "new" copies of duplicated self-interacting proteins. We introduce three new evolutionary parameters, $\mu_{\rm o}$, $\mu_{\rm n}$ and $\mu$, corresponding, respectively, to the probability to conserve the self-link interaction of an "old" or a "new" duplicated gene or the duplication-derived *de novo* interaction between them, Fig. S2.

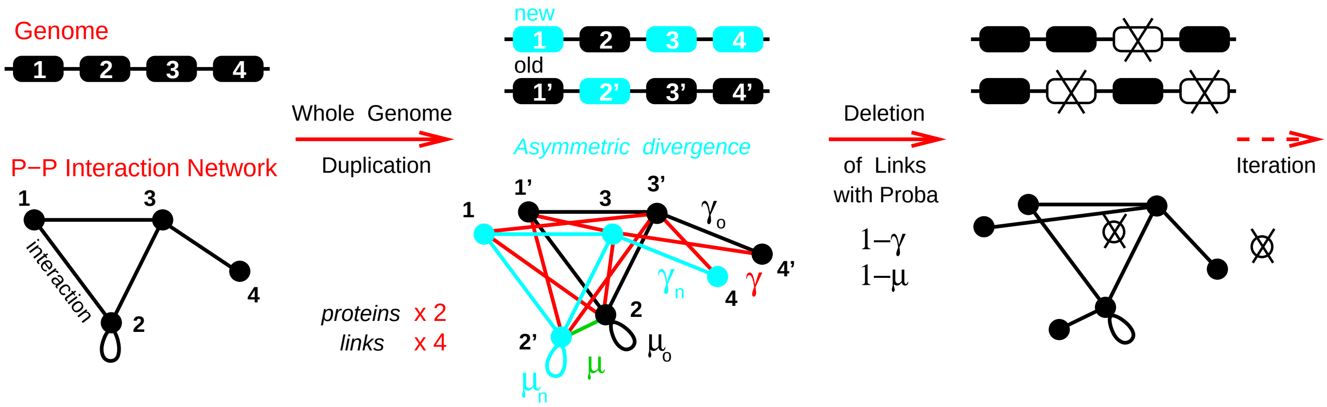


Figure S2. Model of PPI network evolution through whole genome duplication with *asymmetric divergence* of duplicated proteins and including *self-link* interactions.

From a theoretical perspective, we now have to solve two coupled functional recurrences for the generating functions, $F_\ell^{(n)}(x)$ and $F_{n\ell}^{(n)}(x)$, respectively, with and without self-link loops. The global generation function including all network nodes is then simply $F^{(n)}(x) = F_\ell^{(n)}(x) + F_{n\ell}^{(n)}(x)$. Hence, we have,

• Generating function WGD recurrence for the self-link loops, $F_\ell^{(n)}(x)$:

$$F_\ell^{(n+1)}(x) = \big(\mu x + 1 - \mu\big)\Big[\mu_{\rm o} F_\ell^{(n)}\big(A_{\rm o}(x)\big) + \mu_{\rm n} F_\ell^{(n)}\big(A_{\rm n}(x)\big)\Big], \tag{8}$$

where $\mu_{\rm o}$, $\mu_{\rm n}$ and $\mu$ correspond to the selection rates of the loop derived interactions as depicted in Fig. S2 and $A_{\rm o}(x) = (\gamma_{\rm o}x + \delta_{\rm o})(\gamma x + \delta)$ and $A_{\rm n}(x) = (\gamma_{\rm n}x + \delta_{\rm n})(\gamma x + \delta)$, as in the main text, with $\gamma_{\rm o}$, $\gamma_{\rm n}$ and $\gamma$ (resp. $\delta_{\rm i} = 1 - \gamma_{\rm i}$) corresponding to the selection (resp. deletion) rates of the non-loop derived interactions as depicted in Fig. S2.

• Generating function WGD recurrence without self-link loops, $F_{n\ell}^{(n)}(x)$:

$$F_{n\ell}^{(n+1)}(x) = \Big[F_{n\ell}^{(n)}\big(A_{\rm o}(x)\big) + F_{n\ell}^{(n)}\big(A_{\rm n}(x)\big)\Big] + \big(\mu x + 1 - \mu\big)\Big[(1-\mu_{\rm o})F_{\ell}^{(n)}\big(A_{\rm o}(x)\big) + (1-\mu_{\rm n})F_{\ell}^{(n)}\big(A_{\rm n}(x)\big)\Big] \quad (9)$$

with the same notations.

• And the global generating function including all network nodes, $F^{(n)}(x) = F_{\ell}^{(n)}(x) + F_{n\ell}^{(n)}(x)$:

$$F^{(n+1)}(x) = \Big[F^{(n)}\big(A_o(x)\big) + F^{(n)}\big(A_n(x)\big)\Big] + \mu(x-1)\Big[F_{\ell}^{(n)}\big(A_o(x)\big) + F_{\ell}^{(n)}\big(A_n(x)\big)\Big] \quad (10)$$

Note, in particular, that,

- *i)* the evolution of self-link loops, $F_{\ell}^{(n)}(x)$, is not coupled to non-self-interacting proteins, $F_{n\ell}^{(n)}(x)$, while the global network evolution, $F^{(n)}(x)$, is coupled to self-link loops, $F_{\ell}^{(n)}(x)$, if and only if $\mu \neq 0$.

- *ii)* the existence of self-link loops in the PPI network does *not* affect the arguments of *any* generating functions, leading instead to self-link-dependent prefactors in all three generating function recurrences. This implies that the leading term of successive derivatives at $x = 1$ of these generating functions involve successive powers of $\Gamma_{\rm o}$ and $\Gamma_{\rm n}$, as $\Gamma_{\rm o}^k$ and $\Gamma_{\rm n}^k$, where $\Gamma_{\rm o,n} = \partial_x A_{\rm o,n}|_{x=1}$.

Hence, applying the same asymptotic method approach as above readily yields the following asymptotic regimes for *i)* self-interacting proteins and *ii)* global PPI network,

• *i)* we always have $\Delta_\ell = \mu_{\rm o} + \mu_{\rm n}$, for the exponential growth rate of the number of self-link loops, and for scale-free conserved regime, $\Gamma_{\rm o} > 1 > \Gamma_{\rm n}$, $\Delta_\ell = \mu_{\rm o} + \mu_{\rm n} = \mu_{\rm o}\Gamma_{\rm o}^{\alpha_\ell} + \mu_{\rm n}\Gamma_{\rm n}^{\alpha_\ell}$, which defines the power law exponent, $\alpha_\ell$, for the limit degree distribution of self-interacting proteins, $p_{\ell k} \propto k^{-\alpha_\ell - 1}$, $k \gg 1$.

• *ii)* there are two cases for the global network topology in the linear regimes (*i.e.* with same growth rates in terms of node or link numbers):

1- If $\Delta_\ell = \mu_{\rm o} + \mu_{\rm n} < \Gamma_{\rm o} + \Gamma_{\rm n} = \Delta = \Gamma_{\rm o}^{\alpha} + \Gamma_{\rm n}^{\alpha}$, then the network growth rate $\Delta$ is dominated by non-self-interacting proteins, which implies a negligeable effect of self-link loops and no changes from the paper conclusions, in particular $\alpha = \alpha_{n\ell}$, corresponding to the scale-free exponent without self-link loops defined as $\Gamma_{\rm o} + \Gamma_{\rm n} = \Gamma_{\rm o}^{\alpha_{n\ell}} + \Gamma_{\rm n}^{\alpha_{n\ell}}$, as in main text.

2- or else $\Delta_\ell = \mu_{\rm o} + \mu_{\rm n} = \Delta = \Gamma_{\rm o}^{\alpha} + \Gamma_{\rm n}^{\alpha} > \Gamma_{\rm o} + \Gamma_{\rm n} = \Gamma_{\rm o}^{\alpha_{n\ell}} + \Gamma_{\rm n}^{\alpha_{n\ell}}$, then the network growth rate $\Delta$ is dominated by self-interacting proteins, which implies some non negligeable effects of self-link loops but actually *no changes* from the paper main conclusions on network conservation and topological regimes, except for the precise value of the power law exponent $\alpha$ in scale-free regimes, which increases from $\alpha = \alpha_{n\ell}$ to $\alpha_{n\ell} < \alpha < \alpha_\ell$. Note, however, that self-interacting proteins exhibit a larger connectivity exponent $\alpha_\ell$ than the global PPI network, $\alpha < \alpha_\ell$.

Hence, overall, the general conservation and topological properties of PPI networks is actually little affected by the presence of self-link loops, in the asymptotic limits of large PPI networks and large node degrees. As can be seen from the above argument, this is because conservation and topological properties of PPI networks are controlled by the *exponential* increase of their node degrees, $k \to k \times \Gamma_{\rm o,n}$, while the contribution of *de novo* interactions arising from duplicated self-interacting proteins can at most lead to a *linear* increase of node degrees, with a maximum increment of +1 link per duplication event and protein. Thus, although an abundance of self-interacting proteins can significantly affect the evolution of low connectivity proteins, it cannot lead to a change of topological regimes for the highly connected nodes of the PPI networks (*e.g.* from exponential to scale-free node degree distribution or vice versa). Hence, to a first approximation, self-interacting proteins can be simply ignored to establish the asymptotic conservation and topology regimes of PPI network evolution, as we have done in the main text. Note, however, that self-link loops might still be important for the evolution of certain network motifs whose initial emergence might precisely depend on the presence of self-interacting proteins (*e.g.* the triangle motif unless one triangle at least is already present in the initial network).

## References

1. Vázquez A, Flammini A, Maritan A, Vespignani A: **Modeling of protein interaction networks**. *ComPlexUs* 2003, **1**:38–44.
2. Middendorf M, Ziv E, Wiggins C: **Inferring network mechanisms: the Drosophila melanogaster protein interaction network**. *Proc. Natl. Acad. Sci. USA* 2005, **102**:3192–3198.
3. Evlampiev K, Isambert H: **Asymptotic Evolution of Protein-Protein Interaction Networks for General Duplication-Divergence Models**. 2006, *preprint at* http://arxiv.org/abs/q-bio.MN/0611070.
4. Doolittle RF: **The multiplicity of domains in proteins**. *Annu. Rev. Biochem.* 1995, **64**:287–314.
5. Riley M, Labedan B: **Protein evolution viewed through E.coli protein sequences: introducing the notion of a structural segment of homology, the module**. *J. Mol. Biol.* 1997, **268**:857–868.
6. Tatusov RL, Koonin EV, Lipman DJ: **A genomic perspective on protein families**. *Science* 1997, **278**:631–637.
7. Koonin EV, Aravind L, Kondrashov AS: **The impact of comparative genomics on our understanding of evolution**. *Cell* 2000, **101**:573–576.
8. Apic G, Gough J, Teichmann SA: **Domain combinations in archaeal, eubacterial and eukaryotic proteomes.** *J. Mol. Biol.* 2001, **310**:311–325.
9. Bjorklund AK, Ekman D, Light S, Frey-Skott J, Elofsson A: **Domain rearrangements in protein evolution**. *J. Mol. Biol.* 2001, **353**:911–923.
10. Orengo CA, Thornton JM: **Protein families and their evolution-a structural perspective**. *Annual Rev. Biochem.* 2005, **74**:867–900.
11. Fraser HB, Wall DP, Hirsh AE: **A simple dependence between protein evolution rate and the number of protein-protein interactions**. *BMC Evol. Biol.* 2003, **3**:11.
12. Krylov DM, Wolf YI, Rogozin IB, Koonin EV: *Genome Res.* 2003, **13**:2229–2235.
13. Ueda HR, Hayashi S, Matsuyama S, Yomo T, Hashimoto S, Kay SA, Hogenesch JB, Iino M: **Universality and flexibility in gene expression from bacteria to human**. *Proc. Natl. Acad. Sci. USA* 2004, **101**:3765–3769.
14. Lemos B, Meiklejohn CD, Hartl DL: **Regulatory evolution across the protein interaction network**. *Nat. Genet.* 2004, **36**:1059–1060.
15. Lemos B, Bettencourt BR, Meiklejohn CD, Hartl DL: **Evolution of proteins and gene expression levels are coupled in Drosophila and are independently associated with mRNA abundance, protein length, and number of protein-protein interactions**. *Mol. Biol. Evol.* 2005, **22**:1345–1354.
16. Dijkstra E: **A note on two problems in connexion with graphs**. *Numerische Mathematik* 1959, **1**:269–271.
17. Gavin AC, Bosche M, Krause R, Grandi P, Marzioch M, Bauer A, Schultz J, Rick JM, Michon AM, Cruciat CM: **Functional organization of the yeast proteome by systematic analysis of protein complexes**. *Nature* 2002, **415**:141–147.
18. Ho Y, Gruhler A, Heilbut A, Bader GD, Moore L, Adams SL, Millar A, Taylor P, Bennett K, Boutilier K: **Systematic identification of protein complexes in Saccharomyces cerevisiae by mass spectrometry**. *Nature* 2002, **415**:180–183.
19. Gavin AC, Aloy P, Grandi P, Krause R, Boesche M, Marzioch M, Rau C, Jensen LJ, Bastuck S, Dumpelfeld B: **Proteome survey reveals modularity of the yeast cell machinery**. *Nature* 2006, **440**:631–636.
20. Alfarano C, Andrade CE, Anthony K, Bahroos N, Bajec M, Bantoft K, Betel D, Bobechko B, Boutilier K, Burgess E, Buzadzija K, Cavero R, D'Abreo C, Donaldson I, Dorairajoo D, Dumontier MJ, Dumontier MR, Earles V, Farrall R, Feldman H, Garderman E, Gong Y, Gonzaga R, Grytsan V, Gryz E, Gu V, Haldorsen E, Halupa A, Haw R, Hrvojic A, Hurrell L, Isserlin R, Jack F, Juma F, Khan A, Kon T, Konopinsky S, Le V, Lee E, Ling S, Magidin M, Moniakis J, Montojo J, Moore S, Muskat B, Ng I, Paraiso JP, Parker B, Pintilie G, Pirone R, Salama JJ, Sgro S, Shan T, Shu Y, Siew J, Skinner D, Snyder K, Stasiuk R, Strumpf D, Tuekam B, Tao S, Wang Z, White M, Willis R, Wolting C, Wong S, Wrong A, Xin C, Yao R, Yates B, Zhang S, Zheng K, Pawson T, Ouellette BFF, Hogue CWV: **The Biomolecular Interaction Network Database and related tools 2005 update**. *Nucleic Acids Res.* 2005, **33**(suppl1):D418–D424.
